# Comparative study on antibacterial activities and removal of iron ions from water using novel modified sand with silver through the hydrothermal technique

**DOI:** 10.1038/s41598-025-00591-5

**Published:** 2025-05-08

**Authors:** Rashad Al-Gaashani, Mohammad W. Aktar, Khadeeja Abdul Jabbar, Yongfeng Tong, Anas Abutaha, Kamal Mroue, Viktor Kochkodan, Jenny Lawler

**Affiliations:** Qatar Environment and Energy Research Institute (QEERI), Hamad Bin Khalifa University (HBKU), Qatar Foundation, Doha, 34110 Qatar

**Keywords:** Environmental sciences, Materials science

## Abstract

The hydrothermal-calcination technique was used to modify raw sand with silver (Ag) at different weight percentages: 2%, 5%, and 10% using silver nitrate. The raw and sand-coated Ag nanoparticle samples were analyzed using various techniques, including scanning electron microscopy (SEM), transmission electron microscopy (TEM), energy-dispersive X-ray spectroscopy (EDS), X-ray diffraction (XRD), X-ray photoelectron spectroscopy (XPS), Fourier transform infrared spectroscopy (FTIR), and Brunauer–Emmett–Teller (BET) analysis. The antibacterial properties of the raw and modified sand samples were evaluated using the zone of inhibition test and the minimum inhibitory concentration (MIC) method. This study aims to determine the efficiency of sand-coated Ag nanoparticles in removing iron ions and bacteria, specifically *Escherichia coli* (*E*. *coli*) and *Staphylococcus aureus* (*S*. *aureus*), compared to raw sand. The results showed that the Ag content in the modified sand increased proportionally with the silver nitrate concentration. The modification of raw sand with 2% Ag nanoparticles resulted in a significant increase in surface area (85.78%), adsorption pore diameter (138%), and micropore volume (65.65%). The sand modified with 2% Ag exhibited a 31.29% higher removal efficiency and 14.6 mg/g greater adsorption capacity for iron ions than raw sand. A feed pH level between 4 and 10 was found to be optimal for iron removal efficiency (99.95%) using a 1.5 g/L sand-coated with 2% Ag. The maximum adsorption capacity of iron ions obtained in this study was 64 mg/g using sand-coated 2%Ag (dose = 0.2 g/L) at 24 °C in 4 h. Studies utilizing zone of inhibition and MIC methods revealed that the modified sand exhibits strong antibacterial properties against both Gram-positive and Gram-negative strains. In contrast, the raw sand shows no antibacterial activity. All sand-coated Ag nanoparticle samples achieved 100% growth inhibition at 72 µg/mL.

**Supplementary Information:**

The online version contains supplementary material available at 10.1038/s41598-025-00591-5.

## Introduction

Sand is abundant globally, and it can play a major role in water treatment by being used as an adsorbent medium in water filters^1–7^. Modified sand for water treatment was reported such as oxide-coated sand^8^, iron-coated sand^9^ and graphene oxide-coated quartz sand^10^. Sand is very cheap compared to other adsorbents such as activated carbon (AC), silica, zeolite, and kaolinite. In addition, the natural composition and easy availability make it an excellent and sustainable choice for water treatment in many regions. One of the disadvantages of sand as a filtration material is that it is not highly efficient in removing microorganisms from wastewater, and the bacteria grow over time on sand filters. This drastically diminishes the efficiency of the sand filtration process. To minimize biological colonization, the sand can be coated with silver (Ag).

Ag nanoparticles, Ag ions, and Ag composite materials have extensively been studied for their antibacterial and antiviral properties^11–15^. Ag is nontoxic to human cells in low concentrations^16–18^, while Ag nanoparticles show excellent antibacterial activity by binding to microbial DNA and preventing bacterial duplication. Ag nanoparticles are used in medical products like burn dressings and medical device coatings and have the potential to treat drug-resistant bacterial infections^19–21^. Moreover, the use of Ag nanoparticles has shown promise in combating viral infections by inhibiting viral replication and entry into host cells. These unique properties make Ag-based materials a valuable tool in the fight against both bacterial and viral pathogens, offering new possibilities for developing effective treatments and reducing the spread of infectious diseases. Silver-zeolite, a complex of alkaline earth metal and crystal aluminosilicate, is used in Japan for food preservation, disinfection, and decontamination of ceramics. It was reported that Ag cations have no or minimal tissue effect, no antibiotic resistance, and no antibacterial resistance, suggesting Ag and its composite materials could be a useful antibacterial agent for dental materials^22,23^. Its bactericidal properties include absorbing silver ions by bacterial cells and generating reactive oxygen species, which inhibit respiratory enzymes, causing cell damage^22^. Two potential mechanisms by which silver zeolite acts on bacteria were proposed^24^: (1) the bacteria absorb silver ions from the zeolite when they come into contact with it, which damages the bacteria; and (2) the bacterial cells are damaged by the production of reactive oxygen species, which is caused by the inhibition of respiratory enzymes by the silver ions. Kvitek et al.^18^ reported that silver nanoparticles are a more effective antibacterial agent than ionic silver due to their lower toxicity. They effectively suppress bacterial and yeast growth at concentrations of 1–3 mg/L, which are not toxic to human cells or other organisms. This does not pose any risk to humans when used in medical applications.

High iron level is another challenge in the water treatment of ground water. Excessive iron content can be found in groundwater due to corrosion of iron pipes, extensive use of iron-based coagulants, and industrial activities^25^. Although the WHO recommended maximum iron level is 0.3 mg/L, iron concentration in untreated surface water ranges from zero to 50 mg/L^26,27^, while in groundwater levels of the range (2.01–5 mg/L) in Bangladesh^28^ have been observed. High iron levels can cause staining, metallic odor, and blockages in pipes, affecting water pressure and plumbing systems. This can be a significant problem for many people who rely on groundwater as their main source of drinking water^29^. It is important to find effective solutions to this problem to ensure that everyone has access to safe and clean drinking water^26,29^. Exposure to air causes ferrous and manganese to precipitate, giving groundwater a reddish colour. There are various methods available for removing iron from water sources such as electro-coagulation, oxidation, filtration, ion exchange, lime softening, and adsorption. The adsorption process is an efficient and cost-effective method for removing various contaminants, including heavy metals and iron ions, from water^30,31^. Some commonly applied adsorbents for iron ion removal include activated carbon^32–35^, metal oxides^36^, and carbon-based materials^37^.

The main objectives of this study are to evaluate the efficiency of sand modification with silver on the removal of (1) iron ions and (2) bacteria, namely *Escherichia coli* (*E. coli*) and *Staphylococcus aureus* (*S. aureus*) bacteria, from water. The morphology and structure of the modified sands were also studied. The antibacterial activity of the modified sand was evaluated by using zone of inhibition and minimum inhibitory concentration assays. The present study proposes a new strategy for the simultaneous removal of both bacterial contaminants and iron ions through the incorporation of silver nanoparticles positioned onto low-cost modified sand using hydrothermal-calcination method. The modified sand is used with two application functionalities. In contrast to most of the traditional methods which are witheringly targeting microbial removal or metal ion extraction, this study shows a method that can achieve both antibacterial activities as well affinity for iron ion in one material. While the antimicrobial action of silver nanoparticles exhibits a significant increase in the antibacterial activity, the hydrothermal-calcination method for sand modification provides an even high-yield and efficient deposition of silver which represents a new cost-effective way to fabricate low-cost adsorbing materials from naturally available resources that is easily scalable for water purification. This study also provides further investigation into the synergistic nature of modified sand in different concentrations of silver, new manners toward the optimization of its antibacterial and ion removal characteristics. This interaction of both abilities, together with the sustainable utilization of sand for support material, offers a novel and efficient way to purify water in locations under heavy contamination stress such as many areas unaffordable to low-cost treatment technologies, particularly in developing regions.

## Materials and methods

### Materials

Fine brown natural sand was taken from the Qatari desert environment. Silver nitrate (AgNO_3_ ≥ 99%), sodium hydroxide (NaOH, 98.0%), and iron (III) chloride (FeCl_3_, 97%) were purchased from Sigma Aldrich, USA. All aqueous solutions were prepared using deionized water (DIW) with a conductivity of 18.2 MΩ/cm.

### Preparation methods

**Fig. 1:**
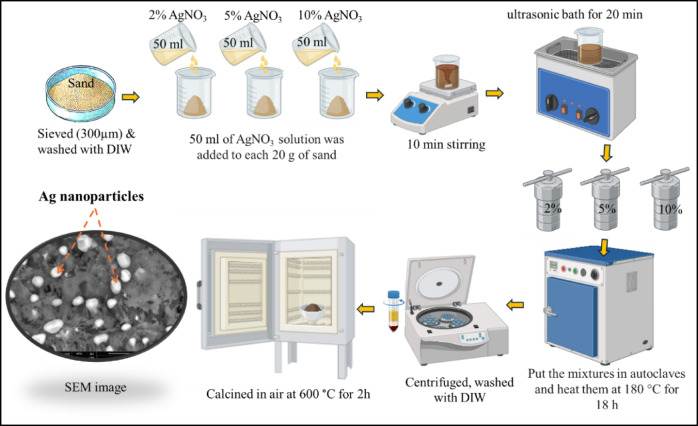
A schematic diagram illustrates the preparation steps for the sand-Ag composite. Some parts are created with BioRender.com.

The sand was first sieved (300 μm) and washed with distilled water. The modification was done by preparing three sand samples with three different AgNO_3_ loadings (2, 5 and 10% wt.). Three portions of sand, each weighing 20 g, were mixed with 50 mL of 2%, 5%, and 10% wt. AgNO_3_ salt solutions. The mixture was stirred magnetically for 10 min and then placed in an ultrasonic bath for 20 min. The mixture was then moved to an autoclave and heated at 180 °C for 18 h. The modified sand with Ag samples were centrifuged, washed multiple times with DIW, and finally calcined in air at 600 °C for 2 h. Figure 1 shows a schematic diagram of the experimental steps to prepare the modified sand with Ag nanoparticles.

### Characterizations

The morphology of the raw and modified sand samples was analyzed using a field emission scanning electron microscope (FE-SEM, QUANTA FEG 650, Thermo Fisher Scientific, USA) and TEM (FEI Talos200X). The elemental composition of the samples was studied by the energy dispersive spectrometer (EDS, Bruker Xflash 6l60, Germany). The broad beam ion milling technique was employed to prepare the cross-section of Ag-coated sand grains for backscattered electron scanning electron microscopy (BSE-SEM) imaging. The grains were adhered to carbon glue and ion milled at 8 kV for 19 h using the Gatan PECS II system. The structural and phase identification of the samples was evaluated by X-ray diffraction (XRD) (Bruker D8 Advance X-Ray diffractometer with Cu-Kα radiation source). The X-ray photoelectron spectroscopy (XPS) measurement is conducted on a Thermo Fisher Escalab 250Xi platform. All XPS measurements are performed in an ultra-high vacuum (7–10 mbar) at room temperature. All binding energies are calibrated to the C-C signal (C1s) at 284.8 eV. The deconvolution of the spectra was conducted with a Voigt-type profile (GL 30) after a proper Shirley background subtraction. Brunauer–Emmett–Teller (BET) analysis was done by an ASAP-2020 surface analyzer to investigate the surface area and porous properties of both raw and modified sand samples. The Fourier transform infrared spectroscopy (FTIR) spectra of the samples were analyzed in transmittance mode utilizing 32 scans per sample and background using Thermo Scientific Nicolet iS50 FT-IR spectrometer to study their functional groups and vibrational modes (see Fig. S1 in Supplementary Information). The zeta potential of both raw and modified sand with 2%Ag was determined using the Zetasizer Ultra from Malvern Panalytical. The measurements were conducted at various pH levels (2, 4, 6, 8, and 10) at room temperature by dissolving the raw and modified sand in deionized water, utilizing the MPT-3 Multi-Purpose Titrator from Malvern Panalytical. Dynamic light scattering was performed with a laser wavelength of 632 nm, and the signal was collected using a backscattering configuration at an angle of 173°.

### Batch adsorption experiments

Iron ion solutions used in this study were prepared from FeCl_3_·6H_2_O and DIW with different concentrations (145, 100, 75, and 30 mg/L).

In a 150 mL flask, the raw and modified sand samples were combined with 25 mL of iron solution to conduct the batch adsorption experiments. The final solution volume was calculated after adjusting the pH values of iron ion solution using either 0.01 M NaOH or 0.01 M HCl solutions. The solutions were then shaken using a Grant OLS Aqua Pro temperature-controlled shaker (Model OLS26, UK) at a rate of 150 rpm. The samples were filtered through a 0.22-micron PTFE membrane syringe filter before being taken for analysis after the adsorption. The iron concentrations were examined with a Hach SL1000 (Iowa, USA) and confirmed by Agilent 5800 Vertical Dual View ICP-OES. The effects of iron concentration, adsorbent type, time, pH, temperature, and adsorbent dose were investigated, as will be detailed in the following sections.

To investigate the effect of sand loading on adsorption, the dose of sand-coated with 2%, 5%, and 10% silver nanoparticles, was varied from 0.2 to 3 g/L at 24 °C, pH of 6.5 ± 0.5, shaking speed of 150 rpm, and contact time of 4 h.

The effect of pH on the adsorption performance of the modified sand was studied at a pH range of 2.5–10.5 at the same other adsorption conditions. The effect of time on the adsorption performance and adsorption kinetics was also investigated after various contact times (9 to 1440 min). The other adsorption conditions were fixed.

Equations (1) and (2) were used to determine the removal efficiency of iron ions from water (%) and the adsorption capacity (Q_e_) in mg of iron ions/g of the raw and modified sand samples:

1 $$Removal\:efficiency\: (\%)=\frac{\left({C}_{o}-{C}_{e}\right)}{{C}_{o}}\times 100$$

2 $${Q}_{e}=\frac{\left({C}_{o}-{C}_{e}\right)\times V}{m}$$

where, *C*_*o*_ and *C*_*e*_ is the initial and final concentration of iron ions in water (mg/L), *V* is the volume of the iron solution (L), and *m* is the raw and modified sand mass (g). Each of the adsorption tests was repeated 3 times and the mean values were reported.

### Antibacterial activity of prepared materials

#### Zone of Inhibition

To attain the exponential phase, cultures of *S. aureus* and *E. coli* were cultivated in Lysogeny broth (LB) broth overnight and then re-cultured for 4 h the following morning. After the suspension was centrifuged to extract the pellet, it was again suspended in a PBS phosphate buffered saline (PBS) solution. Each material, raw sand and sand-coated with 2%, 5%, and 10% silver nanoparticles, was arranged in a circle on the bacterial culture plate, which held the cultures of *S. aureus* and *E. coli*. 10 mg of material was used for each zone of inhibition test. To evaluate each material’s antibacterial activity, the plate was incubated at 37 °C overnight. To see the zone of inhibition, the plate was examined using a lens. Measured surrounding the substance, the zone of inhibition was calculated, and the result was reported in millimeters. The bacterial strain was commercially purchased from ATCC Source and the media used for the zone of inhibition test was Mueller Hinton Agar. The concentration of bacteria was used for the Zone of Inhibition test 1 × 10^8^ CFU/mL S and two replicates were used for each sample.

#### Minimum inhibitory concentration

*E. coli* and *S. aureus* cultures were inoculated in 30 mL of LB Broth and incubated in a shaking incubator at 35–37 °C overnight to allow bacterial growth. The culture was then centrifuged at 3500 rpm for 10 min to obtain pellets, respectively. The pellets were washed and re-suspended with sterile PBS. This process was repeated 3 times. After re-suspending the *E. coli* and *S. aureus* pellets in PBS for the final time, the optical density was then measured to determine the concentration. The *E. coli* and *S. aureus* solution was further diluted in PBS until it reached the concentration of 1 × 10^6^ CFU/mL.

Two separate solutions of the material were prepared: a 1000 ppm solution and a 5000-ppm solution. To prepare the 1000 ppm solution, 5 mg of each material was added to 5 mL of deionized water and vortexed for 10 min to get proper dispersion. The same process was repeated for the 5000-ppm solution, but with 25 mg of each material in 5 mL deionized water. These solutions were then added to the 1 × 10^6^ CFU/mL *S. aureus* and *E. coli* in the following concentrations (shown in Table 1) to evaluate its antibacterial activity. A control group (replacing the 1 × 10^6^ CFU/mL *S. aureus* and *E. coli* solution for LB broth) was created, as well.

**Table 1:** Various concentrations of materials in 1 × 10^6^ CFU/mL *S. aureus* and *E. coli*.

#	*S. aureus* and* E. coil* (1 × 10^6^ CFU/mL)	Material concentration	Amount of material added	DI water	Final material concentration
1	1 mL	0 ppm		4 mL	0 ppm (control)
2		1000 ppm	0.04 mL	1.96 mL	8 ppm
3			0.12 mL	1.88 mL	24 ppm
4			0.36 mL	1.64 mL	72 ppm
5			1.08 mL	0.92 mL	216 ppm
6		5000 ppm	0.648 mL	1.352 mL	648 ppm
7			1.944 mL	0.056 mL	1944 ppm

The above solutions were incubated at 37 °C in a shaking incubator overnight and plated on LB agar plates afterwards. The plates were incubated at 37 °C overnight and the number of colonies were counted the next day.

## Results and discussion

### Morphological and structural study

#### SEM, TEM, EDS, and XRD analysis

**Fig. 2:**
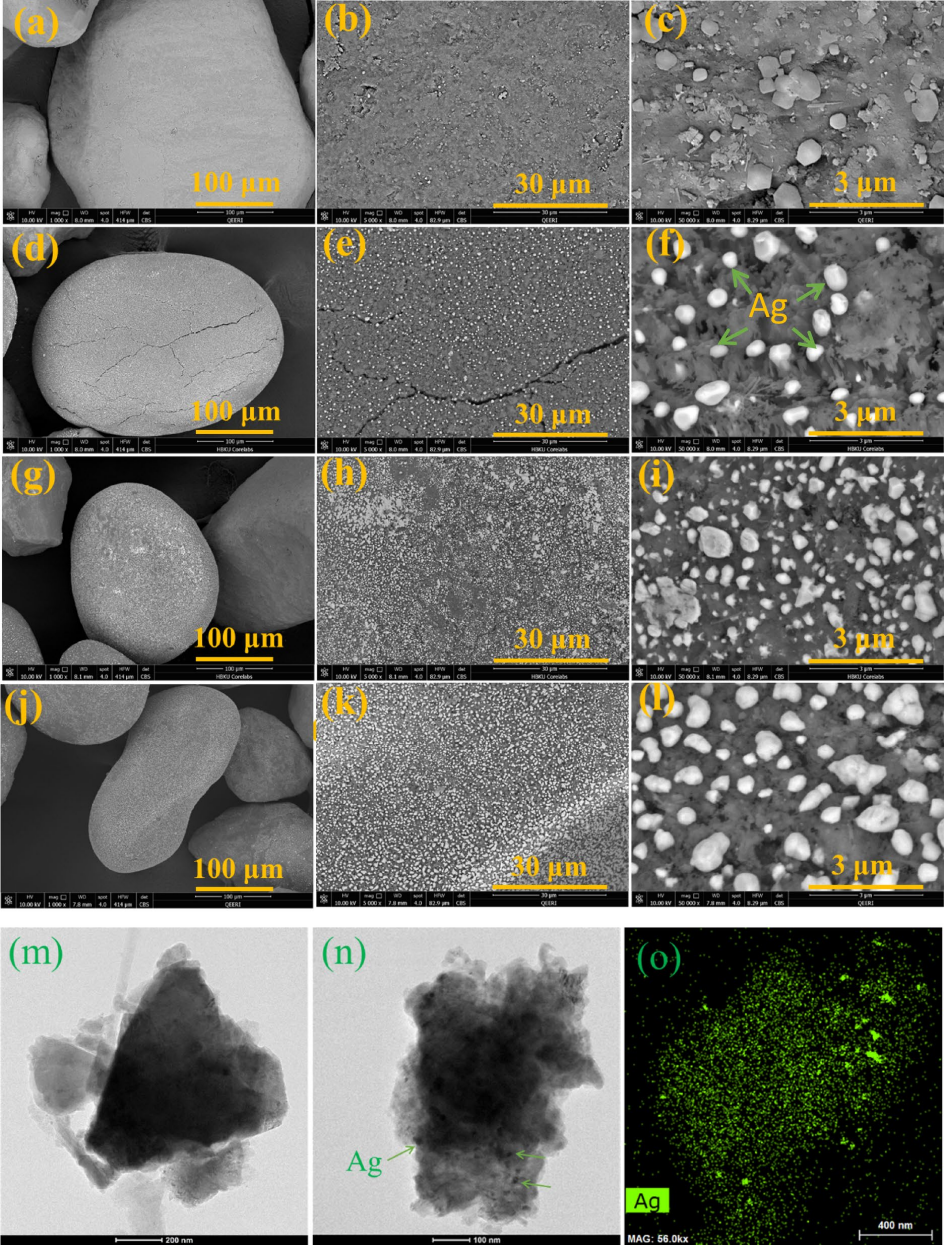
SEM images of sand-coated with silver nanoparticles: (**a**–**c**) images are raw sand, (**d**–**f**) images are sand-coated with 2% Ag, (**g**–**i**) images are sand-coated with 5% Ag, and (**j**–**l**) images are sand-coated with 10% Ag. TEM images of raw sand (**m**), modified sand with 2%Ag (**n**), and its EDS elemental mapping of Ag (**o**). The EDS elemental mapping of all elements is shown in Fig. S2.

**Fig. 3:**
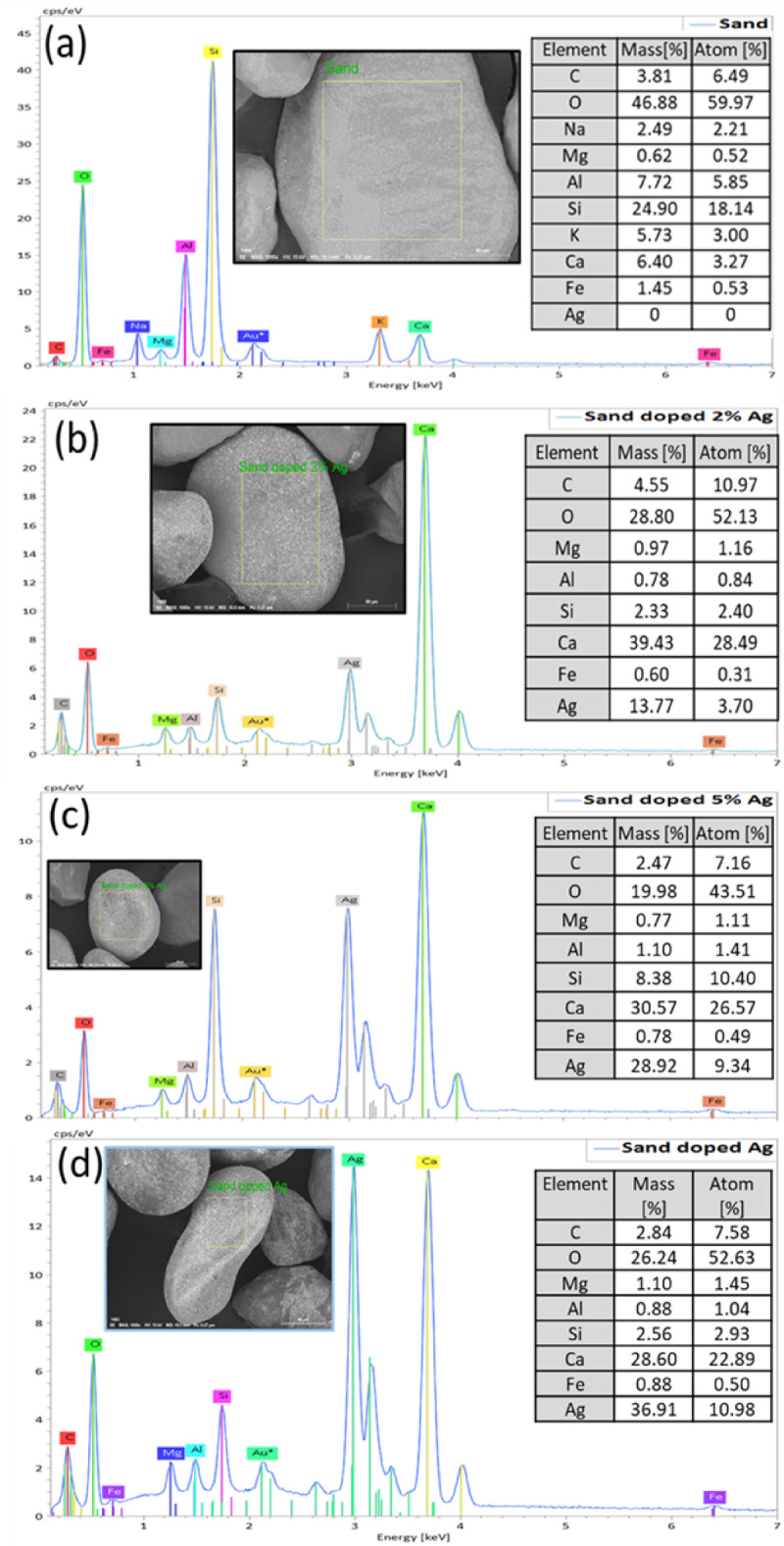
EDS analysis of sand-coated silver nanoparticles: (**a**) is raw sand, (**b**) is sand-coated 2% Ag, (**c**) is sand-coated 5% Ag, and (**d**) is sand-coated 10% Ag.

SEM and TEM analysis is used to study the morphologies of raw and modified sand samples, as seen in Fig. 2a–l. The SEM images of raw sand are shown in Fig. 2a–c, while the SEM images of modified sand with Ag are presented in Fig. 2d–l. The SEM images offer visual evidence of the morphological changes in the sand particles following modification with Ag nanoparticles. These images demonstrate that the addition of Ag nanoparticles modifies the surface structure. Ag nanoparticles grow regularly on the surface of sand grains. This observation suggests that the interaction between sand structure and Ag nanoparticles may play a significant role in modifying the sand. The SEM images also reveal that the size and distribution of the Ag nanoparticles on the sand surface vary depending on the concentration used. This suggests that controlling this factor could potentially lead to tailored modifications of sand properties for specific applications. SEM images (Fig. 2d–l) and EDS (Fig. 3) show that silver content in modified sand increased from 2 to 10% wt. with more Ag salt, indicating successful incorporation of Ag into the sand particles. This successful incorporation of Ag into the sand particles suggests that the modification process effectively enhanced the sand coated with silver content. These findings have significant implications for potential applications in areas such as water purification systems and antibacterial coatings. The TEM images of raw sand and modified sand with 2%Ag and its EDS elemental mapping of Ag are shown in Fig. 2m–o, respectively. The EDS elemental mapping of all elements is shown in Fig. S2. From Fig. 2n, o, it is obvious that Ag nanoparticles are also found inside the ground sand grains, not just on the surface. It is worth noting that studying sand grains using TEM analysis is challenging due to the need for ultra-thin sample preparation, the limited electron transparency of silica, potential charging effects, and low image contrast. SEM analysis is often more practical for analyzing sand morphology.

Figure 3 shows the EDS analysis of raw and modified sand with Ag nanoparticles to determine elemental composition, purity, and effectiveness of the doping process. The elemental composition of raw sand includes C, Na, Mg, Al, Si, Ca, O, and Fe. Once modified with silver (2, 5, and 10 wt%) nanoparticles, the elemental composition of the modified sand samples includes C, Mg, Al, Si, Ca, Fe, O, and Ag. It should be noted that Na and K are present in the raw sand samples, while absent in the modified sand samples. This might be due to the disappearance of salt compounds (Na and K) after washing.

### SEM image analysis of ag nanoparticles on the sand grains

**Fig. 4:**
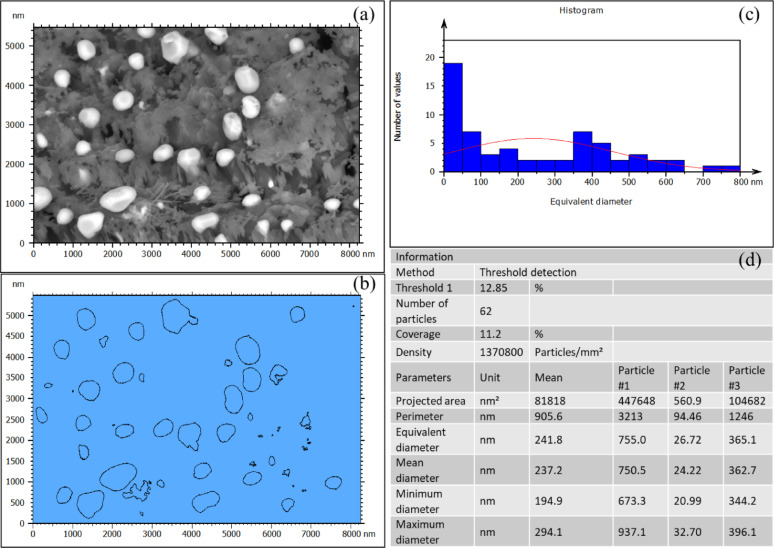
Analysis SEM image of sand-coated with 2% Ag using Mountains software to study the distribution of silver nanoparticles on the surface of a sand grain.

The SEM image of sand-coated with 2% Ag was analyzed using Mountains software from DigitalSurf to study the distribution of silver particles on the surface of a sand grain. The SEM image displays bright silver nanoparticles dispersed over the rough sand surface, Fig. 4a. A processed version of the image highlights the detected particles, outlining their boundaries for size and distribution analysis, Fig. 4b.

The histogram of particle size distribution indicates that most particles fall between 20 and 500 nm, with a considerable portion having an equivalent diameter of less than 50 nm, making them the most frequently occurring size group as shown in Fig. 4c. This suggests that silver deposition primarily results in nanometer-scale clusters rather than larger aggregates.

Statistical data shown in Fig. 4d confirms these findings, showing particle sizes ranging from 20.99 nm (minimum) to 937.1 nm (maximum). The number of detected particles is 62, with a coverage of 11.2% and a density of 1,370,800 particles/mm^2^.

**Fig. 5:**
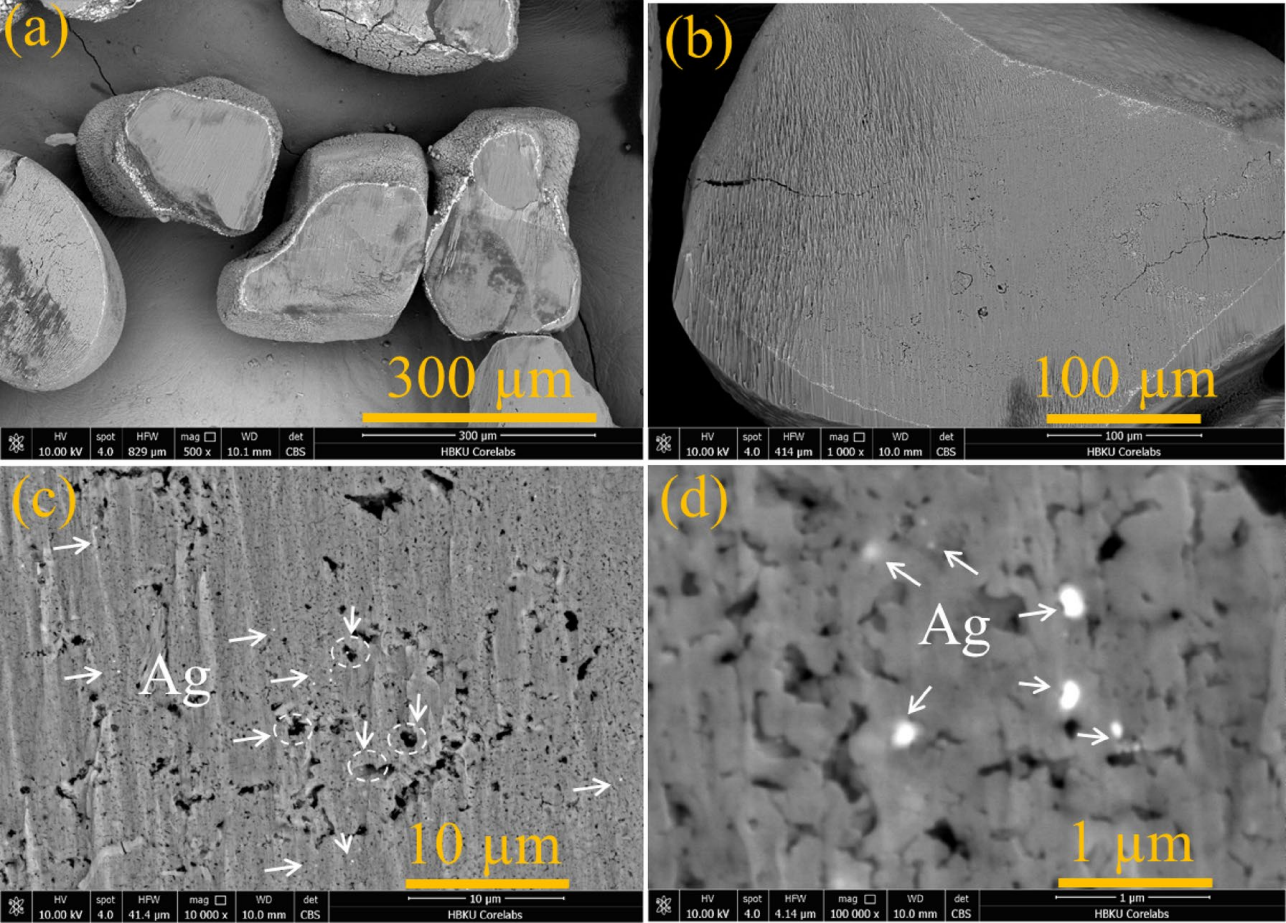
BSE-SEM images of the cross-section of sand grains coated with 2% Ag nanoparticles.

The broad-beam ion milling technique was used to prepare the cross-section of sand grains coated with 2% Ag for SEM with backscattered electron (BSE) imaging. Figure 5a presents a broad overview of the cross-section of sand particles, which exhibit irregular shapes and varying sizes. The bright contrast observed in certain areas suggests the presence of Ag-rich regions, as heavier elements like silver (Ag) appear brighter in BSE-SEM images (a–d). Figure 5b–d shows BSE-SEM images of a single sand grain at different magnifications. These images reveal surface cracks, pores, and distinct textural features. The relatively uniform contrast suggests that Ag is distributed in smaller inclusions rather than forming large deposits. In Fig. 5c, d, the presence of Ag is highlighted, appearing as bright spots due to its higher atomic number. White arrows and circles highlight the presence of diffused silver nanoparticles within the sand matrix and pore spaces, indicating that silver is either mechanically entrapped or chemically integrated into the sand grains. Additionally, the coating on the sand demonstrates an inter-diffusion zone.

**Fig. 6:**
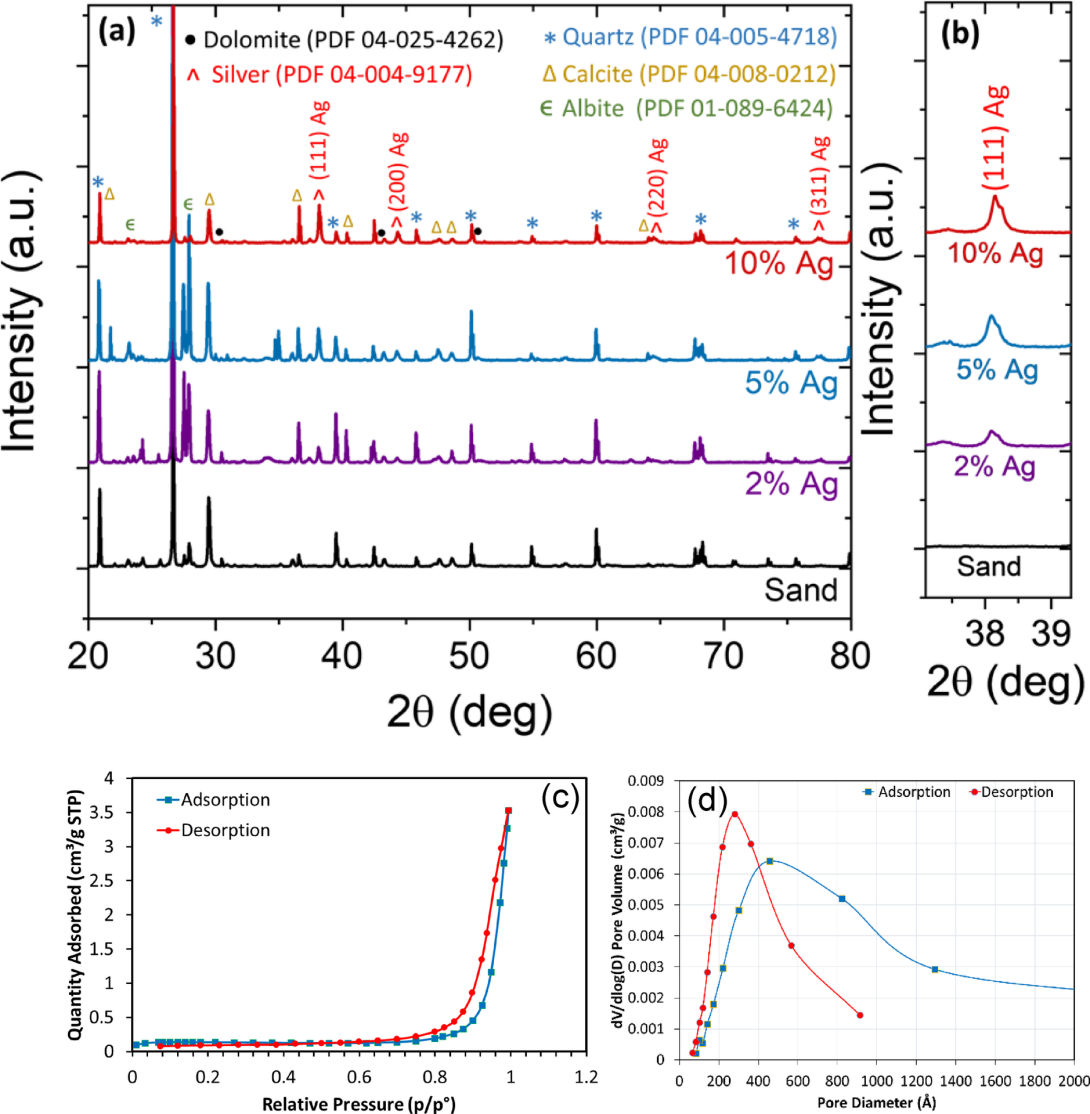
(**a**) XRD patterns for raw sand and sand samples with different Ag doping. (**b**) A magnified view of the XRD diffraction peak for Ag (111). (**c**) Nitrogen adsorption-desorption isotherm of modified sand with 2% silver. (**d**) BJH adsorption and desorption pore size distribution curves.

Figure 6a shows the powder XRD patterns for the pristine sand along with the coated ones with different concentrations of Ag, 2%, 5%, and 10%. The major crystalline phases of the natural compounds in sand were identified such as quartz, calcite, albite, and dolomite, by matching the peaks with data entries from the powder diffraction files (PDF) provided by the International Centre for Diffraction Data (ICDD). All indexed phases were labeled with their respective PDF reference numbers as listed in Fig. 6a. It can be deduced from the XRD patterns that no secondary phases were formed upon Ag loading, indicating that Ag nanoclusters were deposited on sand particles as a separate phase. This observation is consistent with what was observed in SEM images (see Fig. 2) where Ag nanoclusters are observed to be on sand particles. It is evident from the XRD patterns that the diffraction peak attributed to Ag (111) becomes more prominent with loading concentration (see Fig. 6b). The crystallite size for Ag nanoparticles in coated sand samples was calculated using Scherrer equation:

3 $$D=\frac{K\:\lambda}{\beta\: cos\theta }$$

where *D* is the crystallite size; *K* is the Scherrer constant ~ 0.9; $\lambda$ is the X-ray wavelength; $\beta$ is the peak full width at half maximum (FWHM); $\theta$ is the peak position. The Ag crystallite size for all samples was found to be ~ 46–48 nm, indicating that the nonstructural morphologies in the samples are independent of the Ag loading concentration. Table 2 shows some physicochemical properties of raw and sand-coated with 2%Ag. The BET surface area of raw sand is 0.2356 m^2^/g, while the coated sand has a surface area of 0.4377 m^2^/g, representing an increase of approximately 85.75%. This enhancement suggests that Ag nanoparticles contribute to forming a more porous structure, improving the material’s potential for adsorption. Similarly, the micropore area increased from 0.3121 m^2^/g to 0.4605 m^2^/g, reinforcing that silver nanoparticles modify the pore network. The shift in pH_p_*zc* from 2.3 of raw sand to 5.8 after modification by Ag nanoparticles, indicates a shift in surface charge behavior. This suggests that Ag-modified sand exhibits different adsorption properties, influencing its interactions with anionic and cationic contaminants.

**Table 2:** Physicochemical properties of Raw and sand-coated 2%ag.

Physicochemical properties	Raw sand	Sand-coated 2%Ag
BET Surface Area^a^ (m^2^/g)	0.2356	0.4377
Micropore volume (cm^3^/g)	0.000131	0.000217
Micropore area (m^2^/g)	0.3121	0.4605
Adsorption pore diameter (4 V/A) (Å)	180.663	430.243
Desorption pore diameter (4 V/A) (Å)	298.071	284.284
The average particle size^b^ (µm)	100–300	100–300 coated Ag nanoparticles
pH_(pzc)_^c^	6.2^38^ 2.3 this work	5.8

^a^BET Surface Area was measured by using N_2_ adsorption-desorption analysis at 77 K using a Micromeritics ASAP-2020 surface analyzer (USA).

^b^The average partials size was determined using SEM.

^c^pH_(pzc)_: pH at point of zero charge was determined using Malvern Zetasizer Ultra equipment. Adsorption of anions occurs when pH is below the pzc value, while cations are adsorbed when pH is above the pzc value.

Figure 6c displays the nitrogen adsorption-desorption isotherm of sand modified with 2%Ag sample, indicating a characteristic hysteresis loop, suggesting the presence of mesoporous or macroporous materials. Figure 6d shows the pore size distribution curves, indicating a wide pore size distribution, including mesopores (2–50 nm) and possibly macropores (> 50 nm). The shift between adsorption and desorption peaks suggests pore network effects or ink-bottle pores, where desorption occurs at lower pressure due to restricted pore necks^38,39^.

#### Growth mechanism

The growth mechanism of Ag nanoparticles on sand grains will be discussed based on the XRD results and experimental conditions. The XRD analysis indicates that the sand comprises, which contains quartz (SiO_2_), albite (NaAlSi_3_O_8_), calcite (CaCO_3_), and dolomite CaMg(CO_3_)_2_. Sand can adsorb clusters of Ag from aqueous AgNO_3_ that underwent hydrothermal treatment at 180 °C for 20 h, followed by calcination in air at 600 °C for 2 h. During the hydrothermal process, the following reaction can occur:

4 $${\text{A}\text{g}\text{N}\text{O}}_{3}\left(s\right) \mathop{\longrightarrow}\limits_{}^{180^{\circ}\text{C}}{Ag}^{+}\:\left(aq\right)+{{NO}_{3}}^{-}\left(aq\right)$$

During the surface modification, silver ions (Ag⁺) from the decomposition of silver nitrate may also be adsorbed and interact with the sand surface. At high temperature, these silver ions can diffuse into the sand surface and get reduced to form metallic silver nanoparticles within the surface or subsurface layers of quartz, albite, dolomite, and calcite as follows:

5 $${\text{A}\text{g}}^{+}+ {\text{e}}^{-} \mathop{\longrightarrow}\limits_{}^{600^{\circ}\text{C}} Ag\:\left(s\right)$$

The electrons (e⁻) needed for the reduction could come from the thermal environment or other surface interactions. As a result, metallic silver nanoparticles are embedded into the sand structure as shown in Figs. 2 and 5.

It should be noted that most metal nitrates decompose when heated, producing metal oxides. However, silver nitrate decomposes differently, resulting in the formation of elemental silver instead^39^.

#### Thermal decomposition of AgNO_3_

When silver nitrate is heated to around 600 °C, it undergoes thermal decomposition, which results in the breakdown of AgNO_3_ into silver oxide (Ag_2_O), nitrogen dioxide (NO_2_), and oxygen (O_2_) as follows:

6 $$2{\text{A}\text{g}\text{N}\text{O}}_{3}\left(s\right) \mathop{\longrightarrow}\limits_{}^{600^{\circ}\text{C}} {Ag}_{2}O\:\left(s\right)+{2NO}_{2}\left(g\right)+1/2\: O_{2}\left(g\right)$$

However, the Ag_2_O produced in Eq. (6) is unstable at high temperatures and further decomposes into metallic silver and oxygen gas will occur as follows:

7 $${Ag}_{2}O\:\left(s\right) \mathop{\longrightarrow}\limits_{}^{600^{\circ}\text{C}} 2Ag\left(s\right)+1/2\: O_{2}\left(g\right)$$

The Ag_2_O intermediate underwent complete thermal decomposition to produce Ag nanoparticles and O_2_ gas at about 400 °C (673 K)^40^. Ag nanoparticles are finally embedded within the sand structure (quartz, albite, dolomite, and calcite), as illustrated in Figs. 2 and 5.

### XPS study

**Fig. 7:**
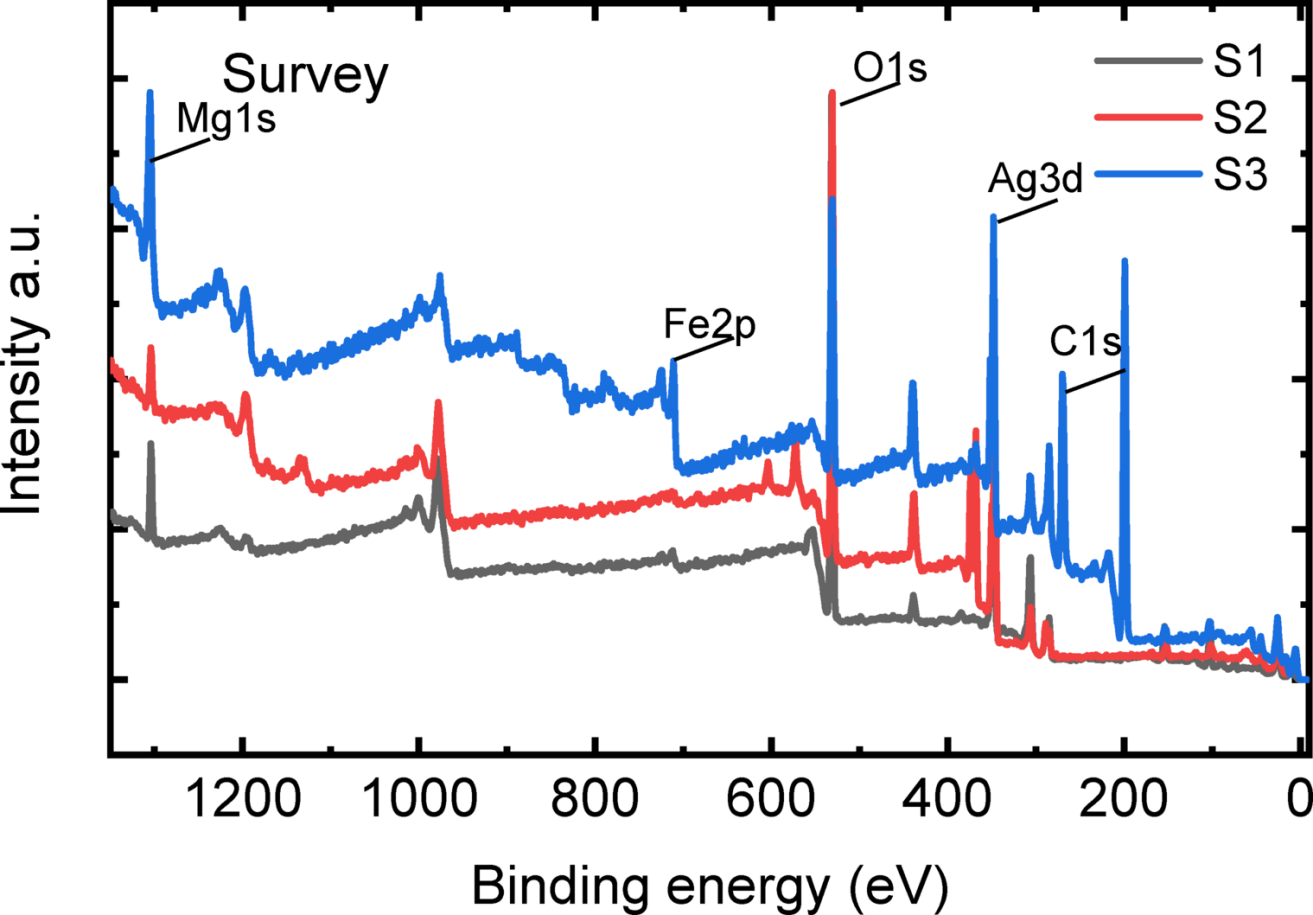
The comparison of the survey spectra of raw sand (S1), sand-coated 2%Ag (S2), and sand-coated 2% Ag after adsorbing iron ions (S3).

Figure 7 shows the comparison of the XPS survey spectra of sand samples before and after doping. The sand sample (black curve) gives elements of C1s, O1s, Ca2p and Si2p, probably related to the CaCO_3_ and SiO_2_. Doping gives the corresponding signal of Ag3d in Sample2 (red curve) and Fe2p in Sample3 (blue curve). Table 3 presents the atomic ratio (%) calculated from the respective high-resolution core levels. It is worth noting that the Ag concentration in Sample 2 was 2.9% and it decreased to 0.4% after Fe doping; meanwhile, despite a small amount of Fe detected in the raw sand sample, the major Fe2p appears mainly in Sample 3 after doping of Fe. The attenuation of Ag and the enhancement of Fe indicate a possible ion exchange process.

**Table 3:** The atomic ratio (%) of the main elements on the surface of the 3 samples.

Sample	Si	C	Ca	Ag	O	Fe	Mg
Raw sand (S1)	12.2	16.7	6.1	0	58.9	1.2	4.8
Sand-coated 2% Ag (S2)	4.3	16.3	15.4	2.9	56.7	1.0	3.4
Sand-coated 2% Ag + iron ions(S3)	4.9	30.7	16.5	0.4	32.7	5.7	9.0

**Fig. 8:**
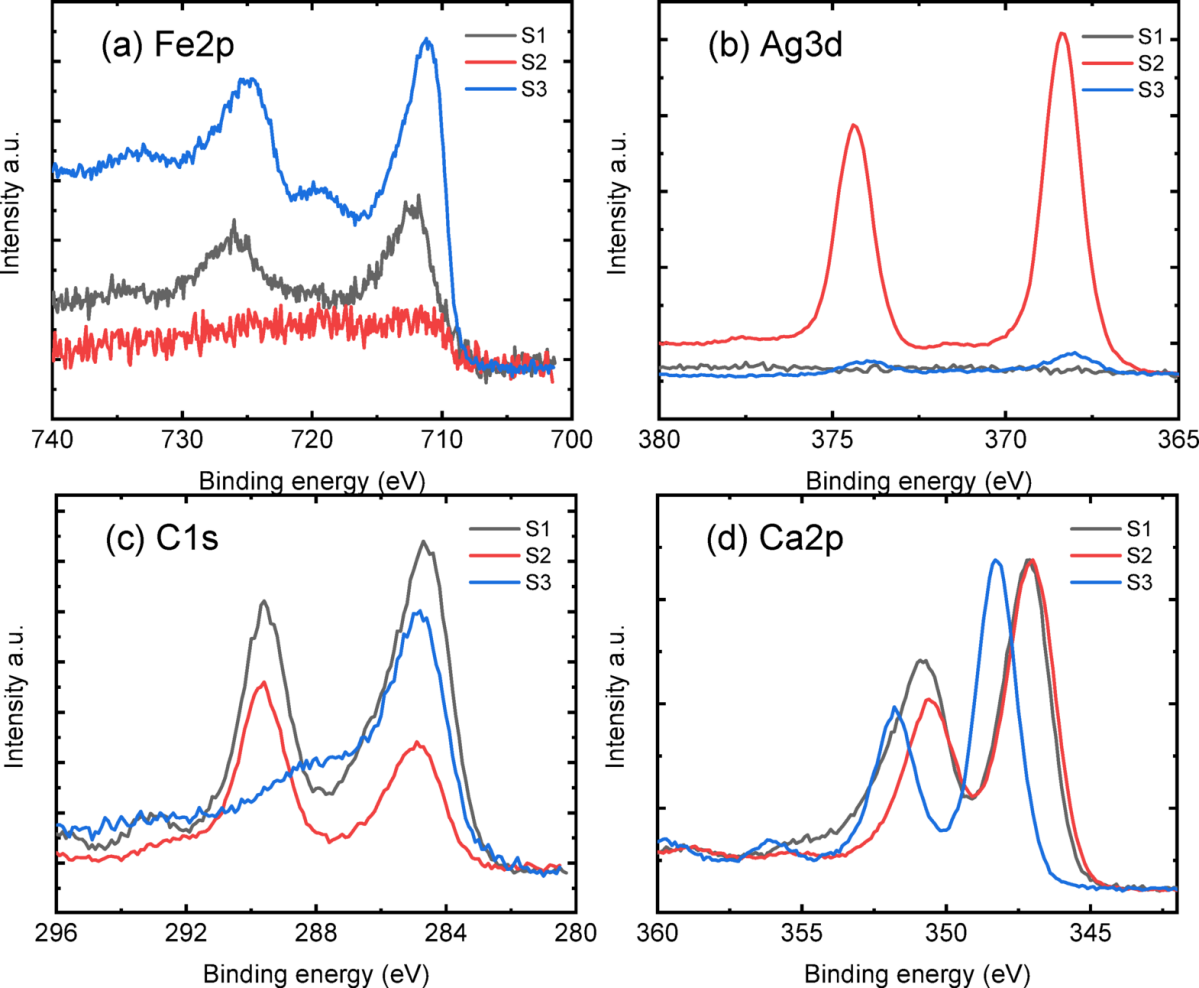
The comparison of the selected core levels of (**a**) Fe2p, (**b**) Ag3d, (**c**) C1s, and (**d**) Ca2p. S1 is raw sand, S2 is sand-coated 2%Ag, and S3 is sand-coated 2%Ag after adsorbing iron ions.

The detailed comparison of high-resolution core levels is given in Fig. 8, where the major signals of Fe2p, Ag3d, C1s, and O1s are selected in (a–d); with the rest given in the supplementary information Figs. S3 and S4. As shown in Fig. 8a, the raw sand sample 1 contains a certain amount of Fe. Doping with Ag significantly attenuates its content in S2; while in S3, the Fe 2p was strongly enhanced after adsorbing Fe ions by the sand-coated 2%Ag sample. The profile of Fe 2p indicates a possible Fe_2_O_3_ state and deconvolution reveals its Fe^2+^, Fe^3+^, and Fe^4+^ at 710 eV, 711 eV, and 713 eV, respectively (see Fig. S3 in the supplementary information). The Ag3d gives a similar (reversed) trend in intensity to the Fe2p signal. The deconvolution of the Ag gives possible Ag^1+^ states, while in sample 3, an Ag chalcogenide bond is given, shown in Fig. 8b, sample 2. The C1s evolution is given in Fig. 8c, where a strong carbonate (~ 289 eV) signal is observed, which becomes more significant in sample 2 after Ag doping but strongly attenuated in sample 3. The loss of carbonate is also proven by the Ca2p in Fig. 8d, where a clear shift is given in sample 3 from the initial carbonate position (~ 347 eV) of the other 2 samples. The comparison of Mg1s, the O1s, and the Si2p core levels is shown in Fig. S4.

### Adsorption results

#### Effect of sand dose on removal efficiency and adsorption capacity

**Fig. 9:**
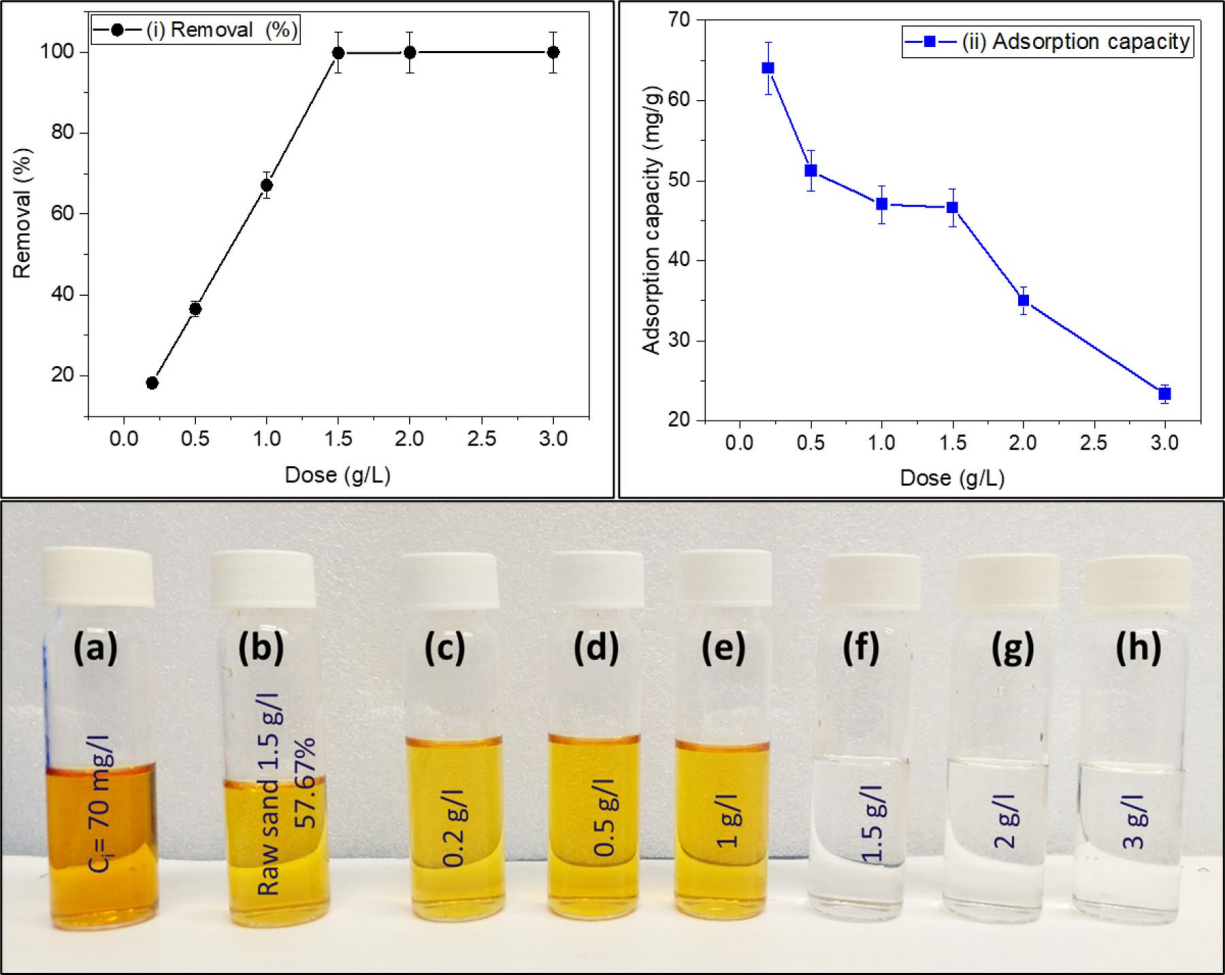
The dose effect of sand-coated 2% Ag on removal % (i) and adsorption capacity of Fe^2+^ (C_o_ = 70 mg/L) from deionized water at RT (24 °C) in 4 h, pH 6.5 ± 0.5 and agitation speed 150 rpm. The photo shows the filtered water after adsorption at various doses (**a**–**h**).

As seen in Fig. 9, the sand-coated with 2% Ag removes 99.85% of Fe^2+^ from the deionized water at a dose loading of 1.5 g/L within 4 h of contact time. The varying colors in the photo indicate the effectiveness of the adsorption process at different doses. This visual representation helps determine the optimal dosage for achieving the desired level of iron ion removal. For water containing 70 ppm of iron, the optimal dosage for effective purification is 1.5 g/L of sand-coated with 2% Ag. This suggests that the material has potential for use in water treatment applications targeting iron removal. However, the optimal adsorption capacity is 64 mg/g, which is achieved at a dosage of 0.2 g/L. At higher dosages, the adsorption capacity decreases as shown in Fig. 9ii, possibly due to the agglomeration of adsorbent particles^41,42^. According to Eq. (2), as the adsorbent loading increases, the adsorption capacity at a given loading decreases because larger loading results in a higher number of adsorption sites while maintaining a constant amount of adsorbate. Thus, using a dosage of 0.2 g/L is recommended for achieving the highest adsorption capacity (64 mg/g).

#### Effect of adsorbent type on iron removal

**Fig. 10:**
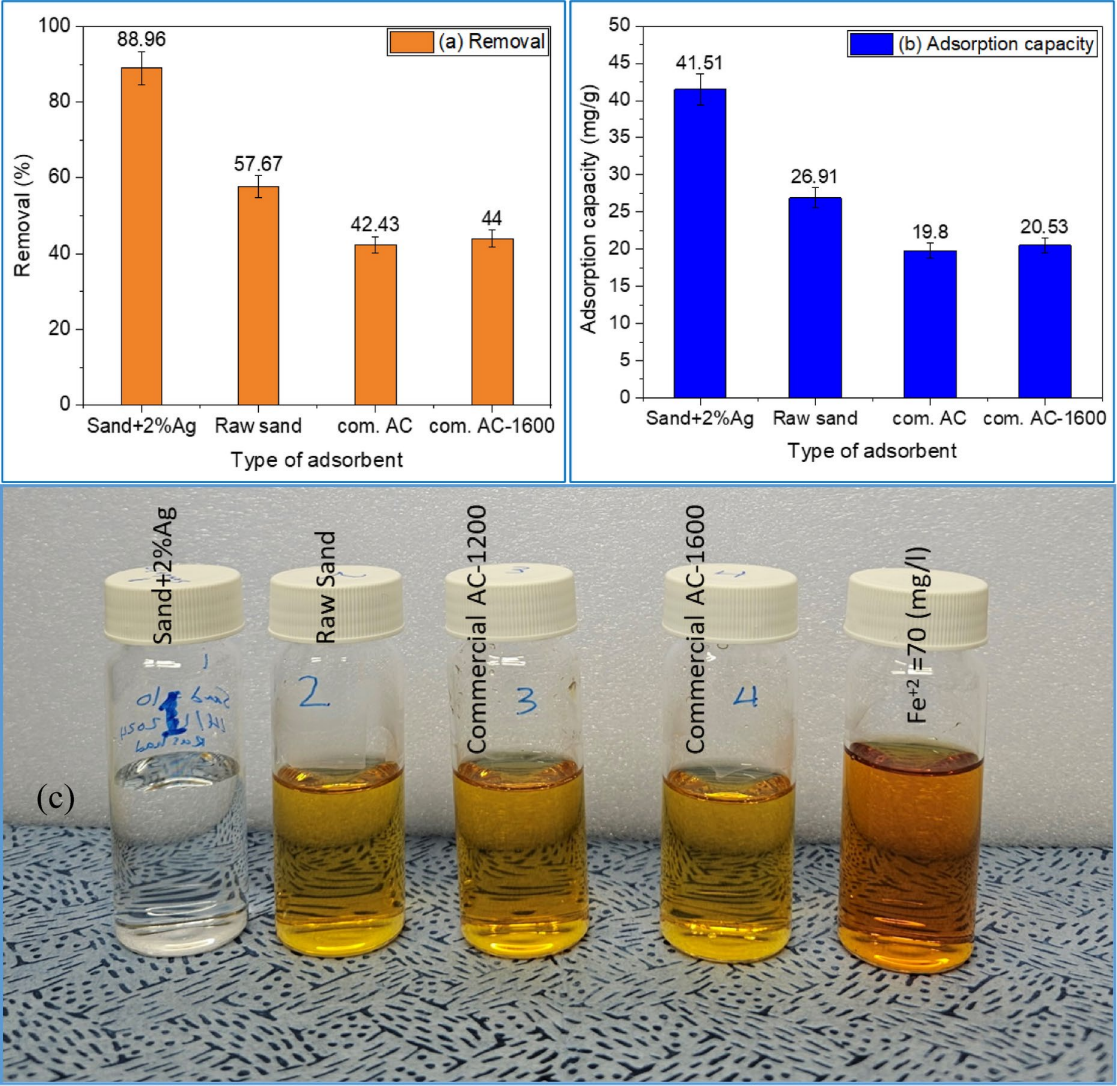
Effect of adsorbent type on removal efficiency (**a**) and adsorption capacity (**b**) of iron ions removal: adsorbent dose is 1.5 g/L, pH 6.5 ± 0.5, C (Fe^2+^) = 70 (mg/L), agitation speed is 150 rpm, RT = 24 ± 0.5 °C for 1 h contact time. The photo shows the filtered water after adsorption by various adsorbents (**c**).

Figure 10 presents a comparative study of various adsorbents, including raw sand, sand-coated with 2% Ag, commercial activated carbon (1100 m^2^/g), and commercial activated carbon (1600 m^2^/g) (Zhulin Activated Carbon Co., Ltd, China), under identical experimental conditions. The results indicate that sand-coated with 2% Ag demonstrated the highest iron ion removal efficiency (88.96%), as shown in Fig. 10a, and the highest adsorption capacity (41.51 mg/g), as displayed in Fig. 10b. This performance was followed by raw sand and activated carbon with a surface area of 1600 m^2^/g. These findings suggest that incorporating silver into sand enhances its adsorption capabilities, potentially making it a sustainable alternative to high-surface-area activated carbon for iron removal from water. Therefore, we have selected it for further study in this work.

Table 4 summarizes the maximum adsorption capacities of iron ions for different adsorbents. After comparing three sand samples coated with 2%, 5%, and 10% Ag, the sand modified with 2% Ag exhibited the best performance in terms of removal efficiency and adsorption capacity.

**Table 4:** The maximum adsorption capacity of iron ions by different adsorbents.

Adsorbent	Dose (g/L)	Initial iron ions concentration (mg/L)	Time (min)	pH	T °C	Maximum adsorption capacity (mg/g)	References
Pecan shell based activated carbon	3	55	90	3	30	41.66	^43^
Manganese oxide (Mn_2_O_3_/Mn_3_O_4_) nanocomposite	2.5	50	62.5	5	RT^a^	18.52	^36^
Modified manganese sand	3.54	Ratio of Fe/Mn = 3.80	720	7.02	RT^a^	0.395	^44^
Aerobic activated sludge	2	50	360	3	20	75.756	^45^
Anaerobic activated sludge	2	50	360	3	20	69.444	^45^
Cow bone charcoal	0. 2	20	20	5.1	25	35	^46^
Sand-coated 2%Ag	0.2	70	240	6.5 ± 0.5	24	64	This study

^a^RT is room temperature.

#### Effect of contact time on iron removal

**Fig. 11:**
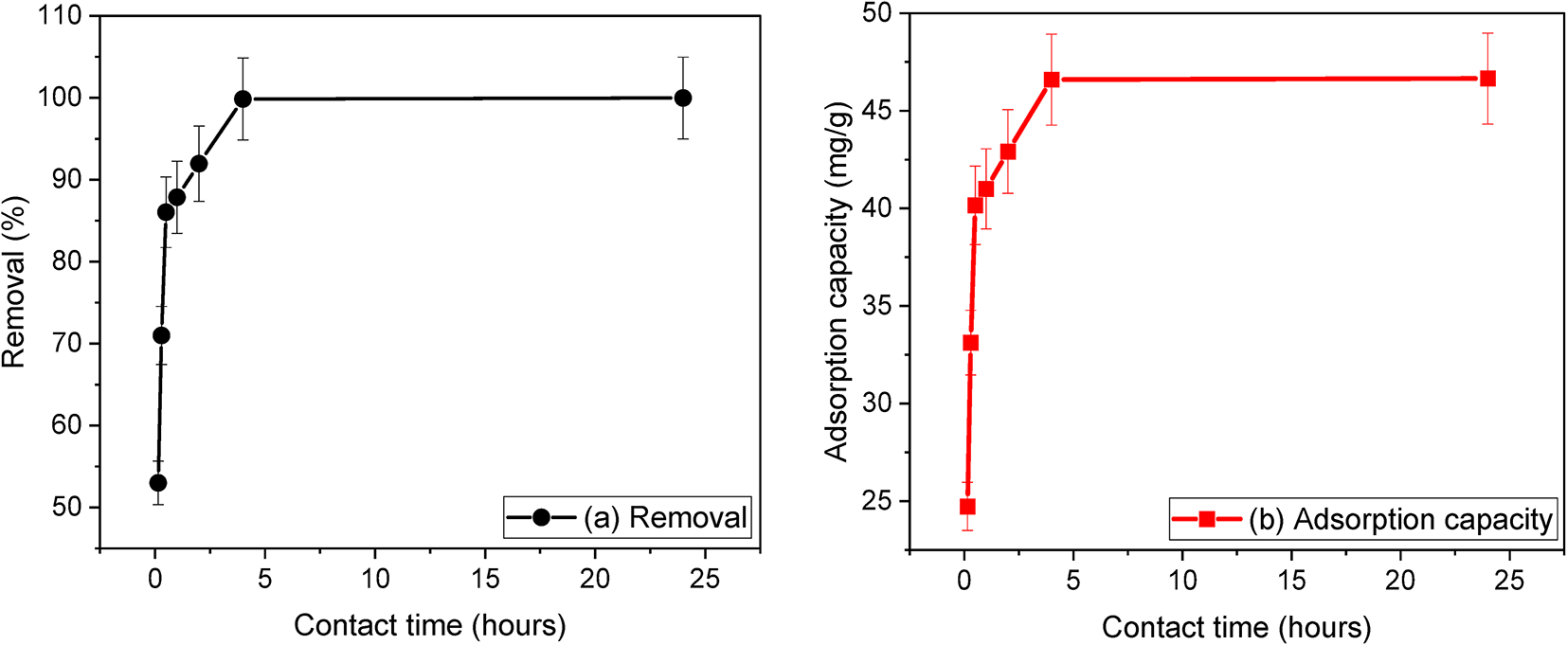
Effect of contact time on (**a**) removal efficiency and (**b**) adsorption capacity of iron ions. Conditions are dose 1.5 g/L (sand-coated with 2% Ag), pH = 6.5 ± 0.5, initial concentration of Fe^2+^ = 70 (mg/L) and agitation speed = 150 rpm at RT = 24 ± 0.5 °C.

The study aimed to determine the effect of contact time on the removal efficiency of iron ions using 1.5 g/L sand-coated with 2% Ag. The contact time varied between 9 min and 24 h while keeping all other parameters constant. The findings illustrated in Fig. 11 indicate that the adsorption process occurs rapidly. After 30 min, the removal efficiency of iron ions reached 86.04%, as demonstrated in Fig. 11a, while the adsorption capacity was measured to be 40.15 mg/g, also shown in Fig. 11b. However, after 4 h of adsorption, the removal efficiency and adsorption capacity increased to 99.85% and 46.60 mg/g, respectively. Therefore, the optimized contact time was determined to be 4 h, and this was used throughout the rest of the study. Increasing the contact time beyond 4 h did not significantly increase the removal efficiency since the adsorption sites on the sand-coated 2% Ag surface had become saturated.

#### Effect of pH and adsorption mechanism

**Fig. 12:**
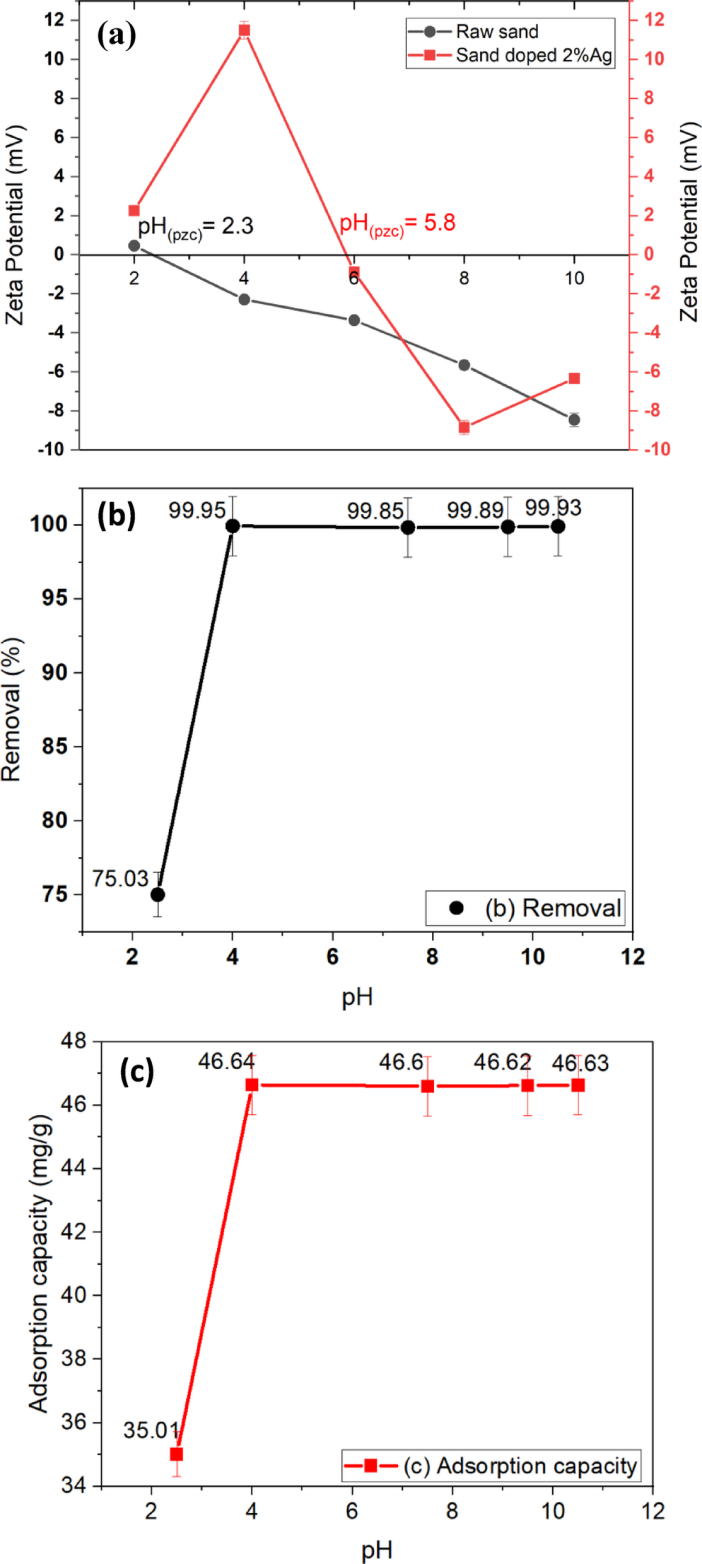
(**a**) Zeta potential of raw sand and sand-coated with 2% Ag. The pH effect on removal efficiency of Fe^2+^ (C_o_ = 70 mg/L) (**b**) and adsorption capacity (**c**) from deionized water at room temperature (24 ± 0.5 °C) for 4 h, with an agitation speed of 150 rpm, using 1.5 g/L of sand-coated with 2% Ag.

The removal efficiency of pollutants by any adsorbent is influenced by factors such as the surface charge of the adsorbent and the solute speciation in water. The point of zero charge (PZC) is the point where the zeta potential is zero, indicating that the amount of positive and negative charges on the material’s surface are balanced. The surface of a material carries a positive charge when the pH value is less than the PZC. However, the surface charge becomes negative when the pH value is above the PZC value. Understanding the PZC is crucial for many applications, including water treatment using adsorption methods. Knowledge of the PZC can help determine the optimal conditions for removing contaminants from wastewater. It can also be useful in the development of new materials with specific surface properties. The PZC of raw sand is found to be 2.3, which has a negative surface charge over a wide pH range. However, sand-coated 2% Ag nanoparticles increased its PZC to 5.8, resulting in a successful modification of raw sand as shown in Fig. 12a. In our new sand-coated 2% Ag nanoparticles, anions will adsorb below the pzc value of 5.8 while cations will adsorb above it.

The effect of pH study on iron removal efficiency and adsorption capacity is presented in Fig. 12b and c, respectively. At pH 4 and above, the highest iron removal rate (99%) and adsorption capacity (46.64 mg/g) were found because it has a negative surface charge that attracts positive iron ions above the PZC. This suggests that to achieve the best iron removal efficiency and adsorption capacity, a pH level that is slightly acidic to neutral must be maintained. On the other hand, reducing the pH to 2.5 led to a marginal drop in iron removal (75%), and adsorption capacity (35.01 mg/g), suggesting that excessive acidity can marginally decline the adsorption efficacy and capacity. This implies that, for the best iron removal efficiency and adsorption capacity, a pH level between 4 and 10 is required. A pH level between 4 and 10 was found to be optimal for iron removal using a 1.5 g/L dose of sand-coated 2% Ag in 4 h. The highest iron removal efficiency at pH 4, despite being below the pH_p_*zc* (5.8), can be attributed to multiple factors. At this pH, the sand surface remains positively charged, promoting the adsorption of negatively charged Fe(III) hydroxy complexes such as Fe(OH)_4_⁻ and Fe(OH)_3_^0^^47^. Additionally, Fe^2^⁺ oxidation to Fe^3^⁺ leads to Fe(OH)_3_ precipitation, enhancing removal efficiency via adsorption and surface complexation^48^. These combined effects explain the observed high removal rate at pH 4^49^.

Iron exists in different oxidation states in aqueous solutions, with ferrous ions (Fe^2+^) and ferric ions (Fe^3+^) being the most common^50^. The presence of various iron species depends on factors such as pH and temperature^51–53^. Fe^2+^ ions are stable in acidic and neutral conditions, while Fe^3+^ ions are stable only in strongly acidic conditions^51,54^. In the pH range of 3 to 6, Fe(OH)_2_ species dominate, transitioning to Fe(OH)_3_ as the solution becomes more basic^55^. Iron (III) chloride species in an aqueous solution can be positive and negative ions: FeCl_2_^+^, FeCl^2+^, FeCl_3_ and FeCl_4_^−^ ions^56^. The PZC of sand-coated with 2%Ag nanoparticles is around pH 5.8. This makes the sand surface suitable for adsorbing positive and negative ion pollutants compared to raw sand with PZC at pH 2.3. Sand has many active sites that can adsorb iron species. When silver nanoparticles are added to the sand surface, they enhance adsorption by forming complexes with iron ions and helping them stick to the sand particles^57^. Silver nanoparticles also could have catalytic activity, which helps to oxidize Fe^2+^ to Fe^3+^^58,59^. This oxidation reaction is facilitated by the presence of oxygen in water. The resulting ferric ions and all iron (III) chloride species can then be adsorbed onto the sand surface.

#### Effect of initial concentration on removal and adsorption capacity of iron ions

**Fig. 13:**
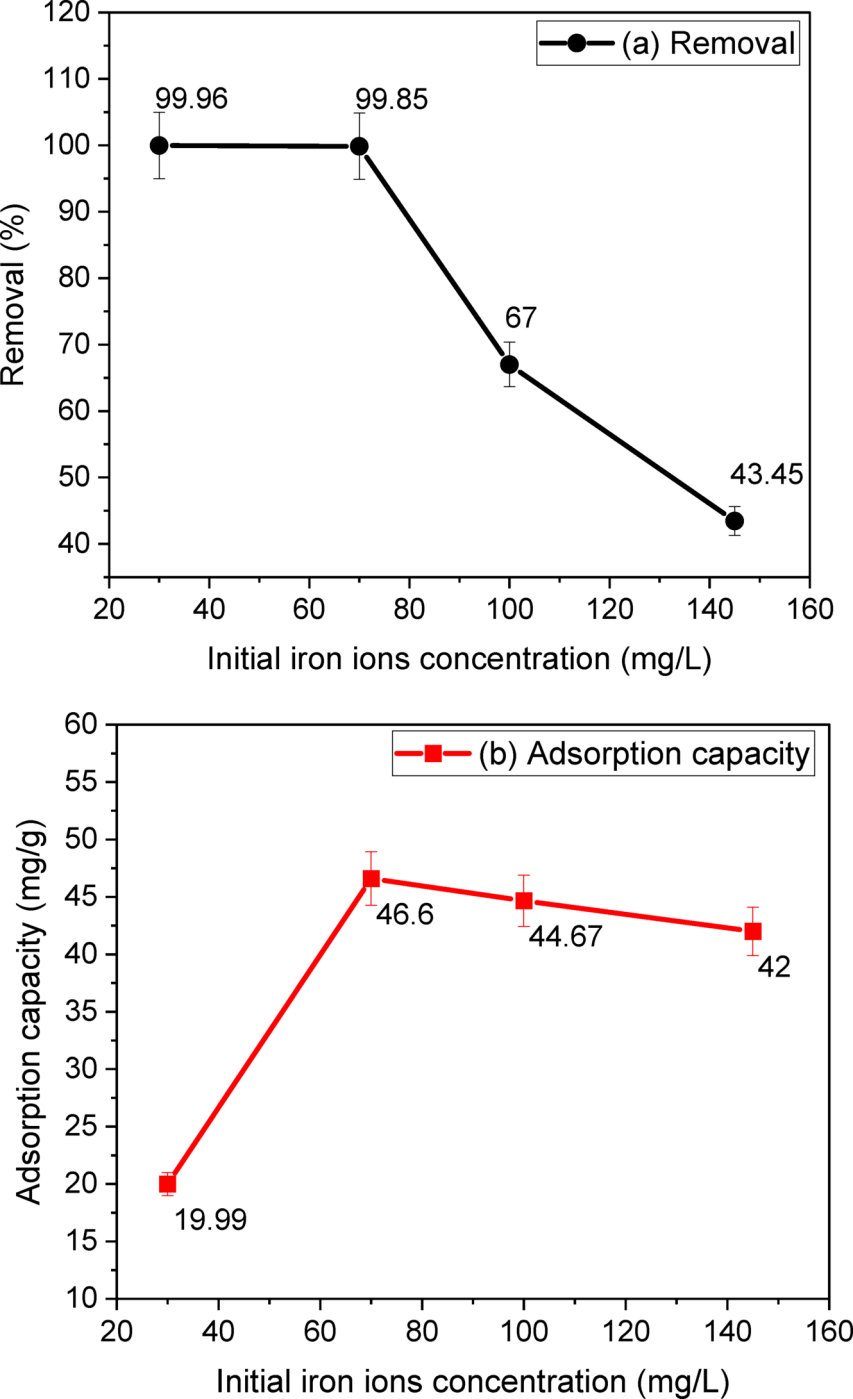
Effect of initial iron ions concentration on iron ions’ removal (**a**) and adsorption capacity (**b**) at 24 °C, pH = 6.5 ± 0.5, agitation speed = 150 rpm, and sand-coated 2%Ag dose = 1.5 g/L in 3 h.

Figure 13 illustrates the effect of the initial concentration of iron ions on their removal and adsorption capacity. The initial concentration from 30 to 145 mg/L effect in the removal of iron ions study shows that an increase in the initial iron ions concentration results in a decrease in the removal of iron ions from 99.96 to 43.45% as shown in Fig. 13a. This result can be attributed to the fact that for a fixed adsorbent dose (1.5 g/L), the total available adsorption sites are limited^36^. Thus, due to the saturation of the adsorbate into the adsorbent and fewer binding sites, with increasing initial iron ion concentration, the removal ratio decreases from 99.96 to 43.45%. This indicates that at higher initial concentrations, the adsorbent becomes overwhelmed and less effective at removing iron ions. It is important to consider the balance between the initial concentration and the adsorbent dose for optimal removal efficiency and adsorption capacity. The optimal adsorption capacity was 46.6 mg/g at an initial concentration of 70% as shown in Fig. 13b. Therefore, we chose the initial concentration of 70% for our study. The efficiency of the adsorption process will be evaluated through isotherm and kinetic studies of the adsorbent in the next work.

### Antibacterial activity of prepared materials

#### Zone of Inhibition

The zone of inhibition and bacterial growth inhibition in aqueous medium were used as model Gram-negative and Gram-positive bacteria, respectively, to examine the antibacterial properties of sand-coated silver nanoparticles against *E. coli* and *S. aureus*. The outcome confirmed the strong bactericidal effects of all the sand-coated silver nanoparticles. The sand treated with 2% silver nanoparticles, as opposed to raw sand, has a better zone of inhibition.

**Fig. 14:**
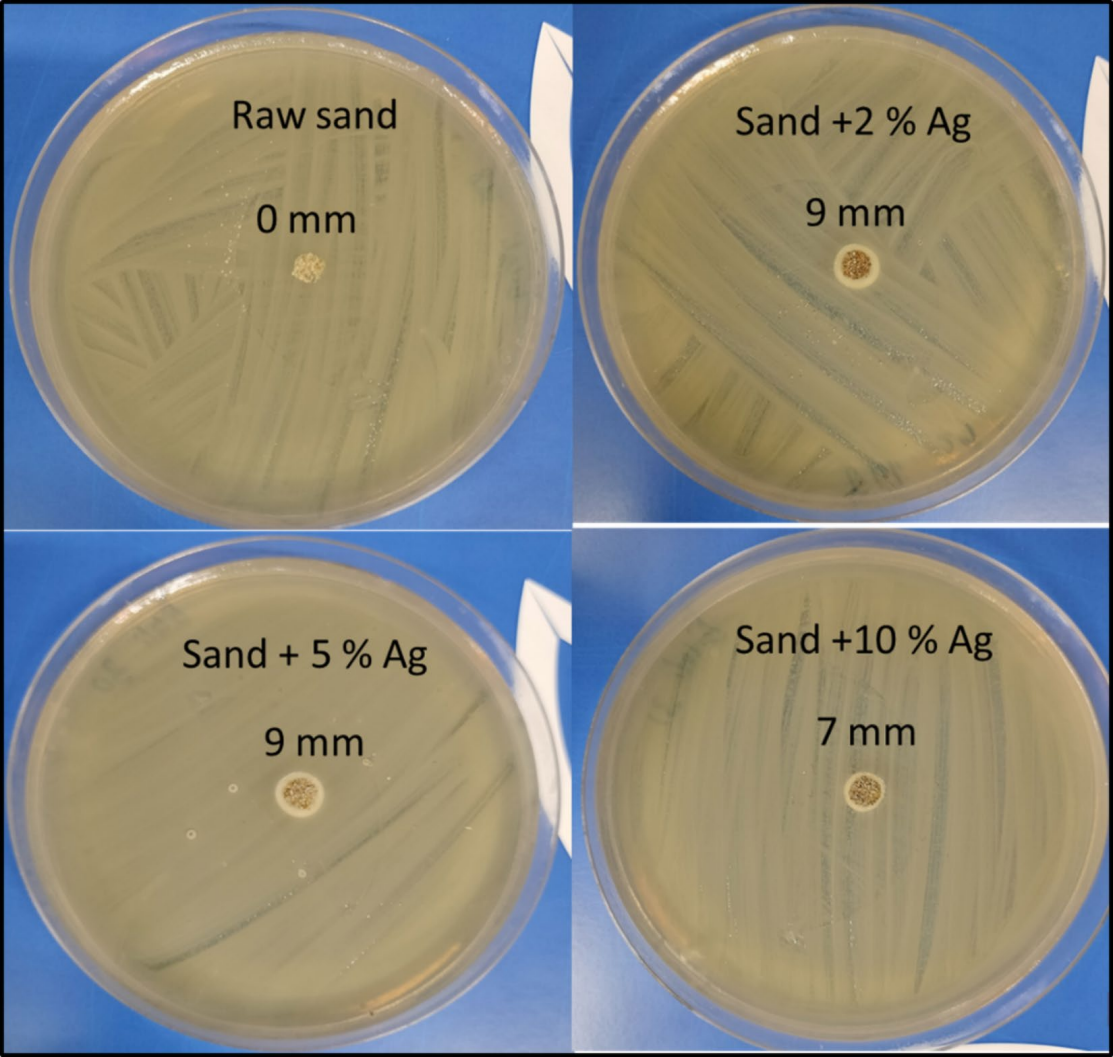
Zone of Inhibition for synthesized material against Gram Negative Bacteria (*E. coli*).

**Fig. 15:**
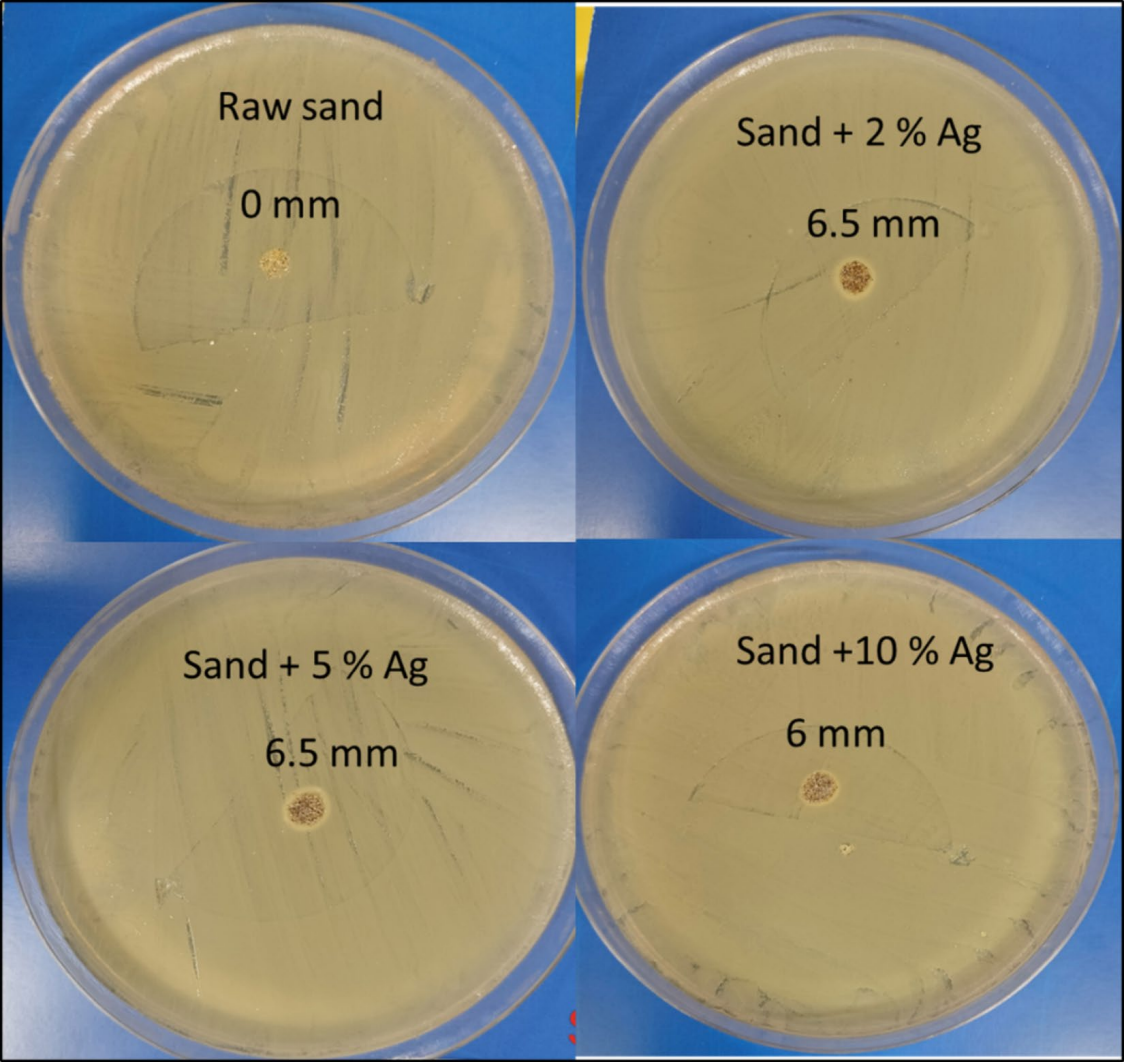
Zone of Inhibition for synthesized material against Gram-Positive Bacteria (*S. aureus*).

According to research presented in the “Materials and methods” section, the antibacterial qualities of raw sand and sand-coated silver nanoparticles against gram-negative (*E. coli*) bacteria have been investigated, and the inhibition zone data are displayed in Fig. 14; Table 5. On the other hand, Fig. 15 shows antibacterial properties towards gram-positive bacteria (*S. aureus*) of (a) raw sand, (b) sand-coated 2 wt% Ag; (c) sand-coated 5 wt% Ag and (d) raw sand-coated 10 wt% Ag.

As seen in Figs. 14 and 15, the raw sand did not display an inhibitory zone with the *E. coli* and *S. aureus* bacteria. Although this result was anticipated, raw sand’s non-antibacterial characteristic may help to explain it. Conversely, Fig. 14; Table 5 display distinct zones of inhibition against *E. coli* bacteria for sand-coated Ag nanoparticles at different Ag concentrations. However, Fig. 15; Table 5 demonstrated the significant zones of inhibition for sand-coated Ag nanoparticles with different silver concentrations against *S. aureus* bacteria. The antibacterial activity of sand-coated Ag nanoparticles appears to be greater against *E. coli* bacteria than against *S. aureus* bacteria. Due to the existence of an outer membrane (peptidoglycan-porin proteins) that shields the cell wall, *E. coli* bacteria are, however, often more resistant to antibiotics. Many antibiotics are unable to pass through this membrane because lipopolysaccharides (LPS) form a barrier that keeps them from doing so^60,61^.

An increase in Ag loading in the composite materials causes the inhibition zone’s diameter to expand to 9.0 mm for *S. aureus* and *E. coli*. The coated Ag’s bactericidal action on *S. aureus* and *E. coli* cells is what caused the sizable inhibitory zones to be seen. Since silver is not very harmful to people, research on the antibacterial qualities of materials containing silver is highly valued^11,62^. The exact processes behind these materials’ bactericidal effect have not yet been determined. According to some research, silver nanoparticles may attach to and enter *E. coli* bacterium, harming the bacterial cell^63^. It has also been proposed that certain bacterial enzymes may bind to silver ions, which would then disrupt the respiratory chain and kill the bacterial cell^64^. Rendering reactive oxygen species in the presence of free oxygen and silver, which can attack and damage a bacterial cell’s outer membrane and ultimately cause it to die, was suggested as a typical mechanism of bactericidal action of silver-coated materials. Another possibility is that a bacterial cell may sorb silver ions, disrupting DNA replication in the process^62^.

The presence of Ag ions suggests that the bacteria are either killed by the ions’ strong electrostatic interactions with the negatively charged bacterial cell membrane or by the activation of internal reactive oxygen species. Sand-coated Ag nanoparticles demonstrated a significant antibacterial effect when membrane function was disrupted and biomacromolecules were oxidized. According to Figs. 12 and 13, and Table 5, the highest zone of inhibition value was 9 mm for both *S. aureus* and *E. coli*, observed for the sand-coated 2% Ag nanoparticles sample.

**Table 5:** Antibacterial composite materials and their Preparation methods and effects on Inhibition zones for *E. coli* and *S. aureus* bacteria.

Antibacterial composite nano-materials	Preparation methods	Zones of inhibition for* E. coli* (mm)	Zones of inhibition for* S. aureus* (mm)
Raw sand		0	0
Sand-coated 2% Ag nanoparticles	Simple impregnation–calcination	9	9
Sand-coated 5% Ag nanoparticles	Simple impregnation–calcination	9	7.5
Sand-coated 10% Ag nanoparticles	Simple impregnation–calcination	7	6.5

#### Minimum inhibitory concentration

We further examined the antibacterial efficiency of sand-coated with Ag nanoparticles against *E. coli* and *S. aureus* at different concentrations (8, 24, 72, 216, 648 and 1944 µg/mL) after overnight incubation. Figure S5 displays the control group of *S. aureus* and *E. coli*.

Figures S6–S9 and Table 6 show the amount of CFUs loss of *E. coli*, while Figs. S10–S13 and Table 7 display the amount of CFUs loss of *S. aureus* as the concentrations of sand-coated Ag nanoparticle suspensions increase. The corresponding photographs show a decrease in the number of colonies with increasing concentration. For *E. coli* cells, bacterial inhibition was more pronounced at 24 µg/mL. Doping with 2% Ag nanoparticles showed greater suppression of cell growth compared to sand-coated 5% and 10% Ag nanoparticles at all doses. The growth inhibition of 100% was achieved using sand-coated Ag nanoparticles at 72 µg/mL. For *S. aureus* cells, bacterial inhibition was more pronounced at 24 µg/mL. Sand-coated 2% Ag nanoparticles showed slightly higher cell growth inhibition than sand-coated 5% and 10% Ag nanoparticles for all concentrations. At 72 µg/mL, sand-coated 5% and 10% Ag nanoparticles reached growth inhibition of 100%. In the case of both bacteria, it was observed that as the concentration of sand-coated Ag nanoparticles increased, a lower inhibition rate was observed in sand-coated 10% Ag nanoparticles as compared to sand-coated 5% and 2% Ag nanoparticles. The reason for this might be the uniform distribution and shape of the Ag nanoparticles, which is shown in Fig. 2.

**Table 6:** Amount of *E*. *coli* colonies in various concentrations of Raw sand, sand-coated 2, 5, and 10% ag nanoparticles.

#	*E. coli* (1 × 10^6^ CFU/mL)				
	Concentration (ppm)	Amount of CFUs (raw sand)	Amount of CFUs (sand-coated 2% Ag nanoparticles)	Amount of CFUs (sand-coated 5% Ag nanoparticles)	Amount of CFUs (sand-coated 10% Ag nanoparticles)
1	0 (control)	239	239	239	239
2	8	239	238	235	235
3	24	169	0	9	125
4	72	151	0	0	0
5	216	0	0	0	0
6	648	0	0	0	0
7	1944	0	0	0	0

**Table 7:** Amount of *S. aureus* colonies in various concentrations of Raw sand, sand-coated 2, 5 and 10% ag nanoparticles.

#	*S. aureus* (1 × 10^6^ CFU/mL)				
	Concentration (ppm)	Amount of CFUs (raw sand)	Amount of CFUs (sand-coated 2% Ag nanoparticles)	Amount of CFUs (sand-coated 5% Ag nanoparticles)	Amount of CFUs (sand-coated 10% Ag nanoparticles)
1	0 (control)	265	265	265	265
2	8	210	247	209	198
3	24	171	0	13	95
4	72	125	0	0	0
5	216	0	0	0	0
6	648	0	0	0	0
7	1944	0	0	0	0

The relative cell viability of *E. coli* and *S. aureus* is displayed in Tables 8 and 9 as the concentrations of sand-coated Ag nanoparticle suspensions rise, respectively. Figures S6–S13, display the related photographs, which demonstrate how the number of colonies decreases as concentration increases. Growth inhibition of 100%, 96.23%, and 47.70% for *E. coli* was observed at a concentration of 2%, 5%, and 10% Ag nanoparticles at 24 µg/mL. Bacterial suppression was more noticeable for *S. aureus* cells at 24 µg/mL, resulting in cell viability decreases of 4.91%, and 35.85% for sand-coated 2%, 5%, and 10% Ag nanoparticles, respectively. In comparison to sand-coated 5% and 10% Ag nanoparticles, 2% Ag nanoparticles inhibited cell proliferation more strongly across all doses. All sand-coated with Ag nanoparticles attained 100% growth inhibition at 72 µg/mL.

**Table 8:** Relative cells viability of *E. coli* in various concentrations of Raw sand, sand-coated 2, 5 and 10% ag nanoparticles.

#	*E. coli* (relative cells viability %)				
	Concentration (ppm)	Amount of CFUs (raw sand)	Amount of CFUs (sand-coated 2% Ag nanoparticles)	Amount of CFUs (sand-coated 5% Ag nanoparticles)	Amount of CFUs (sand-coated 10% Ag nanoparticles)
1	8	100%	99.58%	98.33%	98.33%
2	24	70.71%	0%	3.77%	52.30%
3	72	63.18%	0%	0%	0%
4	216	0%	0%	0%	0%
5	648	0%	0%	0%	0%
6	1944	0%	0%	0%	0%

**Table 9:** Relative cells viability of *S. aureus* in various concentrations of Raw sand, sand-coated 2, 5 and 10% ag nanoparticles.

#	*S. aureus* (relative cells viability %)				
	Concentration (ppm)	Amount of CFUs (raw sand)	Amount of CFUs (sand-coated 2% Ag nanoparticles)	Amount of CFUs (sand-coated 5% Ag nanoparticles)	Amount of CFUs (sand-coated 10% Ag nanoparticles)
1	8	79.25%	93.21%	78.87%	74.72%
2	24	64.53%	0	4.91%	35.85%
3	72	47.17%	0	0	0
4	216	0	0	0	0
5	648	0	0	0	0
6	1944	0	0	0	0

Previous studies on concentration-dependent antibacterial activity for graphene oxide and reduced graphene oxide at 80 µg/mL for *E. coli* shown 91.6% and 76.8% growth inhibition, respectively^62^. Another study showed the growth inhibition for *E. coli* at 99% after 24 h of incubation using 21%Ag@MXene^65^. Previous studies about the zone of inhibition on *E. coli* and *S. aureus* were found 8 mm and 12 mm using Ag-ZnO/activated carbon^66^, 9.7 mm and 8.3 mm using ZnO-doped Ag nanoparticles^67^ and 8 mm and 4 mm using Ag-loaded ZnO nanoparticles^68^, respectively.

All the microbiological experiments revealed that sand-coated 2% Ag nanoparticles exhibited the highest antibacterial properties against both gram-positive and gram-negative bacteria compared to other sand-coated Ag nanoparticles.

### The challenges and limitations

Table 10 summarizes the main challenges and limitations of this study and proposes solutions for future research aimed at overcoming these challenges to improve practical applications.

**Table 10:** Summarizes the challenges and suggests potential solutions for future research.

Challenges and limitations	Proposed solution
Large-scale application challenges: Scaling up the modified sand with Ag using the hydrothermal method for real-world applications could face cost and efficiency issues	Optimize the synthesis process to reduce costs and improve efficiency Study alternative preparation methods and materials Testing alternative materials to silver such as ZnO and CuO to reduce the cost and maintain antibacterial properties
Long-term stability: The durability of the silver coating over time is uncertain, with potential leaching concerns	Conduct long-term stability studies under various environmental conditions Develop protective coatings or binding techniques to improve adhesion Reducing the percentage of silver
Regeneration and Reusability: The material’s effectiveness after multiple uses needs further study	Explore regeneration techniques such as chemical or thermal treatment to restore activity Conduct reusability tests to determine the optimal number of cycles Reusing modified sand with Ag in the production of building materials, thus preserving the foundations of buildings from biological corrosion

## Conclusions

Batch adsorption studies were conducted to investigate the effect of sand-coated with Ag at different adsorbent doses (0.2–3.0 g/L), initial solution pH (2.5–10.5), contact time (9–1440 min), initial iron ions concentration (70–145 mg/L), and temperature (25–45 °C) on the adsorptive removal of iron from synthetic water. The findings of the adsorption tests indicate that the use of modified sand infused with silver demonstrates a higher removal efficiency of 31.29% and an increased adsorption capacity of 14.6 mg/g for iron ions compared to raw sand. A pH range of 4 to 10 was found to be ideal for iron removal, achieving 99.95% when using a 1.5 g/L dose of sand-coated with 2% Ag in 4 h. In comparison to raw sand, modified sand exhibits antibacterial activity, making it more effective in preventing bacterial growth. The maximum adsorption capacity for iron ions was 64 mg/g, achieved experimentally using sand-coated 2% Ag nanoparticles. This was demonstrated by the zone of inhibition and minimum inhibitory concentration (MIC) studies, which showed that the modification process enhances the sand’s ability to inhibit the growth of both Gram-positive and Gram-negative strains. In the microbiological tests, the sand coated with 2%Ag showed the highest antibacterial properties against both gram-positive and gram-negative bacteria, outperforming the sand-coated with 5 and 10%Ag. Sand-coated with Ag can be recycled after adsorption and used in the production of building materials. For instance, it can be used to make bricks that are resistant to bacterial corrosion and are suitable for use in wet foundations and all types of buildings. This process not only helps to reduce waste but also provides a sustainable solution for the construction industry. Future work will focus on studying the isotherm, kinetic, and thermodynamic investigations of the adsorbent and exploring further optimization of the adsorption process. The recyclability test will be conducted in the production of building materials.

## Electronic supplementary material

Below is the link to the electronic supplementary material.

Supplementary Material 1

## Data Availability

The datasets used and/or analyzed during the current study available from the corresponding author on reasonable request.
