# Supplementary material for: Comparative study on antibacterial activities and removal of iron ions from water using novel modified sand with silver through the hydrothermal technique
Source: Sci Rep. 2025 May 8;15:16097. doi: 10.1038/s41598-025-00591-5 (PMC12062301; doi:10.1038/s41598-025-00591-5)
Supplement: Supplementary file 1 — Supplementary Material 1 [file 41598_2025_591_MOESM1_ESM.docx]

# **Supplementary Information**

**Comparative Study on Antibacterial Activities and Removal of Iron Ions from Water Using Novel Modified Sand with Silver Through the Hydrothermal Technique**

Rashad Al-Gaashani^⁎^, Mohammad W. Aktar, Khadeeja Abdul Jabbar, Yongfeng Tong, Anas Abutaha, Kamal Mroue, Viktor Kochkodan, Jenny Lawler

*Qatar Environment and Energy Research Institute (QEERI), Hamad Bin Khalifa University (HBKU), Qatar Foundation, 34110 Doha, Qatar*

****Corresponding author.*** *Tel: 0097430571456. E-mail:* [*ralgaashani@hbku.edu.qa*](about:blank)

## **FTIR study**

Fig. S1 displays the FTIR spectra of three samples: raw sand (a), sand-doped with 2% silver (b), and sand-coated with 2% silver and adsorbing iron ions (sand-coated 2% Ag + Fe) (c). The FTIR spectra provide information about the chemical properties and structures of materials based on their functional groups and vibrational modes. By comparing the position, intensity, and shape of the absorption peaks for each sample, we can evaluate the effect of silver and iron doping on the chemical properties of sand. In the spectrum of pure sand (a), the main peaks are observed around 1000 cm^−1^ and 800 cm^−1^, corresponding to the Si-O-Si asymmetric and symmetric stretching vibrations, respectively. These peaks are characteristic of the silica matrix in sand. For sample (b), which is sand coated with 2% Ag, the spectrum shows a slight shift in the Si-O-Si stretching vibrations, indicating that the incorporation of Ag ions affects the silica network. Si–O–H stretching band is observed around 3400 cm⁻¹. The Ag-O bending modes can be observed at (500 cm^−1^) ^1^. The absence of distinct Ag peaks in the FTIR spectra is expected due to the nature of metallic silver and its interaction with infrared radiation. Metallic silver (Ag⁰) does not exhibit strong FTIR absorption because it lacks permanent dipole moments, which are essential for infrared-active vibrations ^2^. If silver exists as nanoparticles or metallic clusters on the sand surface, their FTIR signals may be weak or undetectable ^3^. For silver oxides (Ag₂O, AgO), characteristic Ag–O stretching vibrations typically appear in the 400–600 cm⁻¹ range ^1,2^. However, these peaks could overlap with Si–O and Si–O–Si stretching vibrations (450–1100 cm⁻¹), making them difficult to distinguish^3^. FTIR is not the best technique for detecting metallic silver, so we employed alternative methods like XRD, EDS, and XPS. The Si-O-Si peaks are broadened, indicating a more disrupted silica network due to the presence of both Ag and iron ions as shown in Fig. S1 (c). New peaks around 600 cm^−1^ and 450 cm^−1^ are observed, which can be assigned to Fe-O vibrations, indicating the formation of iron oxide or the interaction of iron ions with the silica matrix. The presence of both Ag and iron leads to a more complex spectrum, reflecting the combined effects of these dopants on the sand structure. A broad carbonate band is observed at about 1450 cm⁻¹ in raw sand and sand coated with 2% Ag samples in the FTIR spectrum. However, in the sand-coated with 2% Ag after the adsorption of the iron ions sample, the carbonate bands appear diminished, suggesting partial interaction or possible dissolution due to the iron ions solution during adsorption time as shown in Fig. S1 (c).


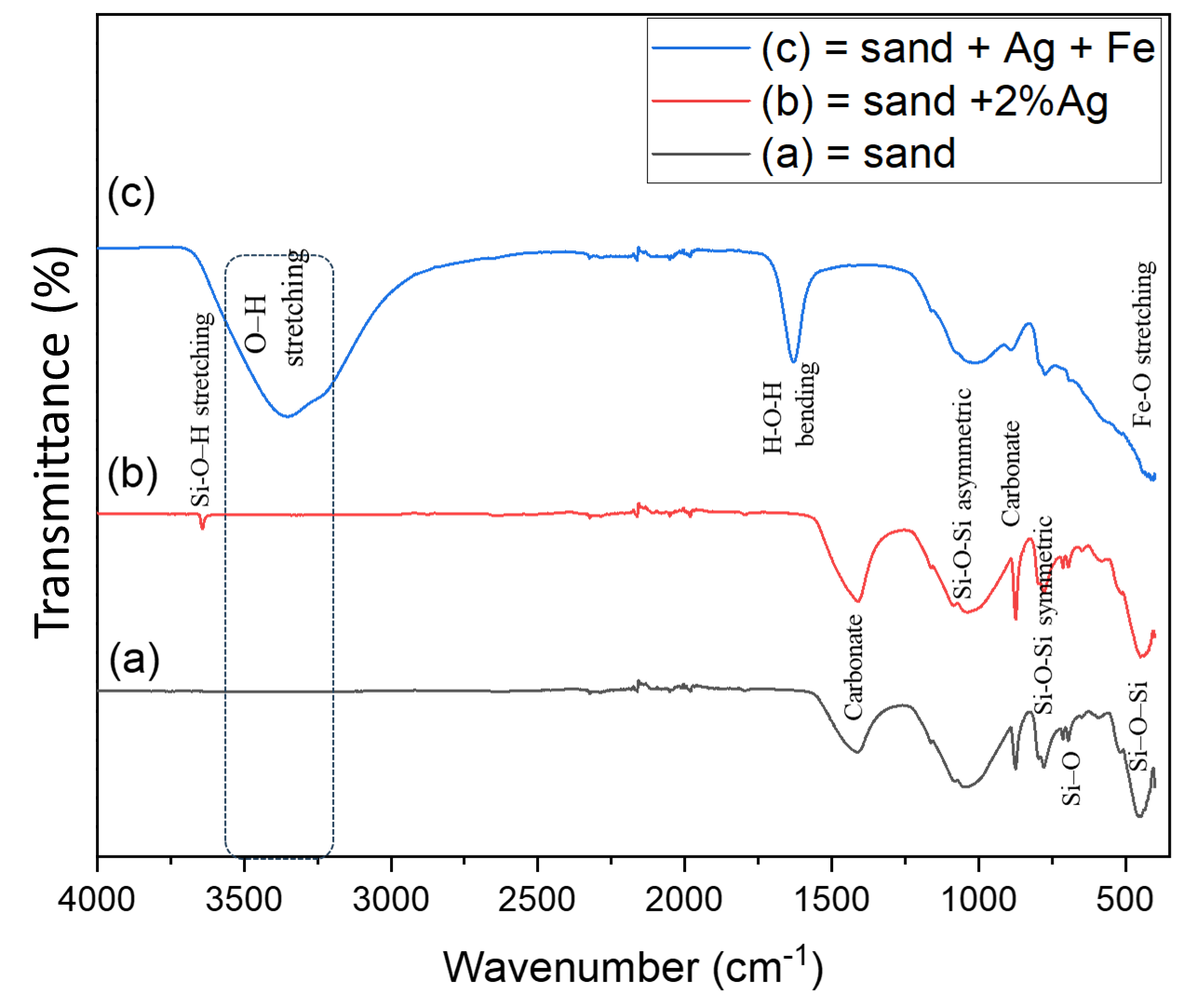


Fig. S1. FTIR of sand (a), sand-coated 2%Ag (b) and sand-coated 2%Ag after adsorption of iron ions (c).


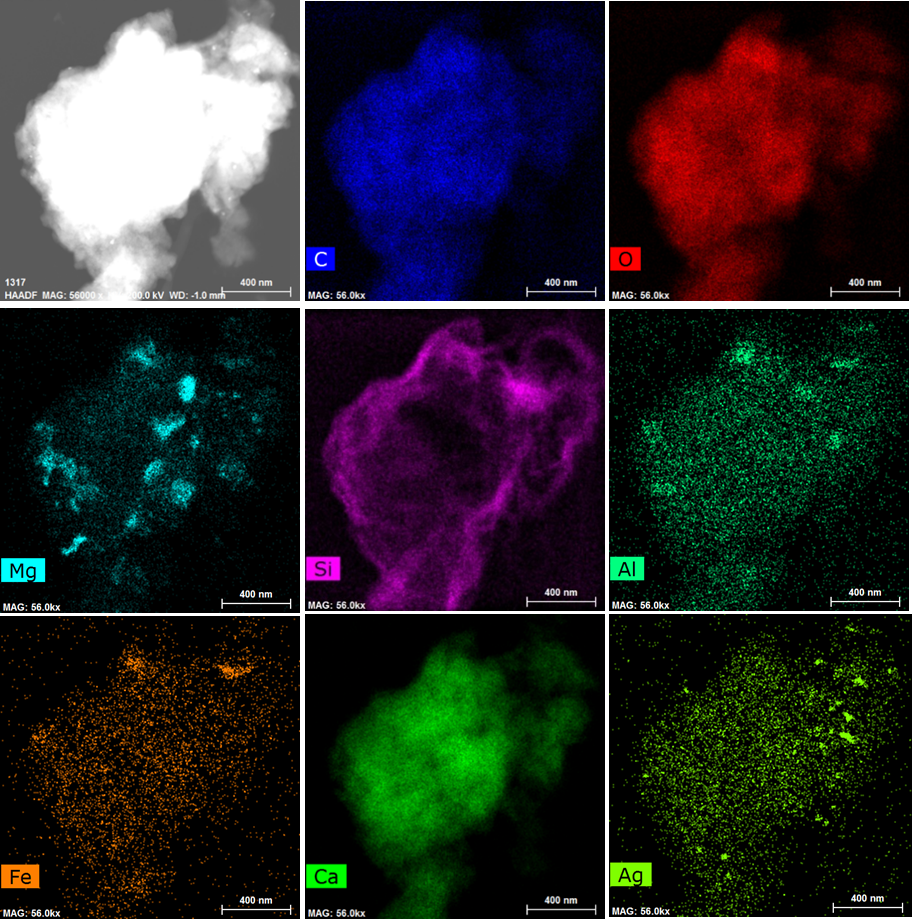


Fig. S2. The EDS elemental mapping of sand-coated 2% Ag sample. The primary elements identified are carbon (C), oxygen (O), magnesium (Mg), silicon (Si), aluminum (Al), iron (Fe), calcium (Ca), and silver (Ag).

Fig. S3. deconvolution of the selected HR core levels of Fe2p, Ag3d, C1s, Ca2p, O1s, and Si2p, respectively.

Fig. S4. The comparison of Mg1s, the O1s, and the Si2p core levels.

*
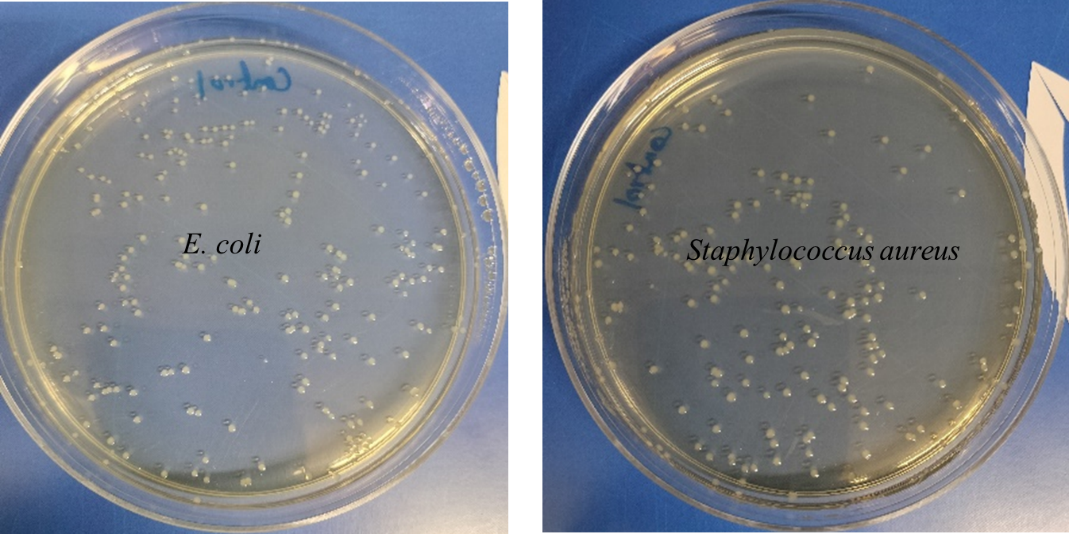
*

Fig. S5. *E.coli & S. aureus* Control Group.

*
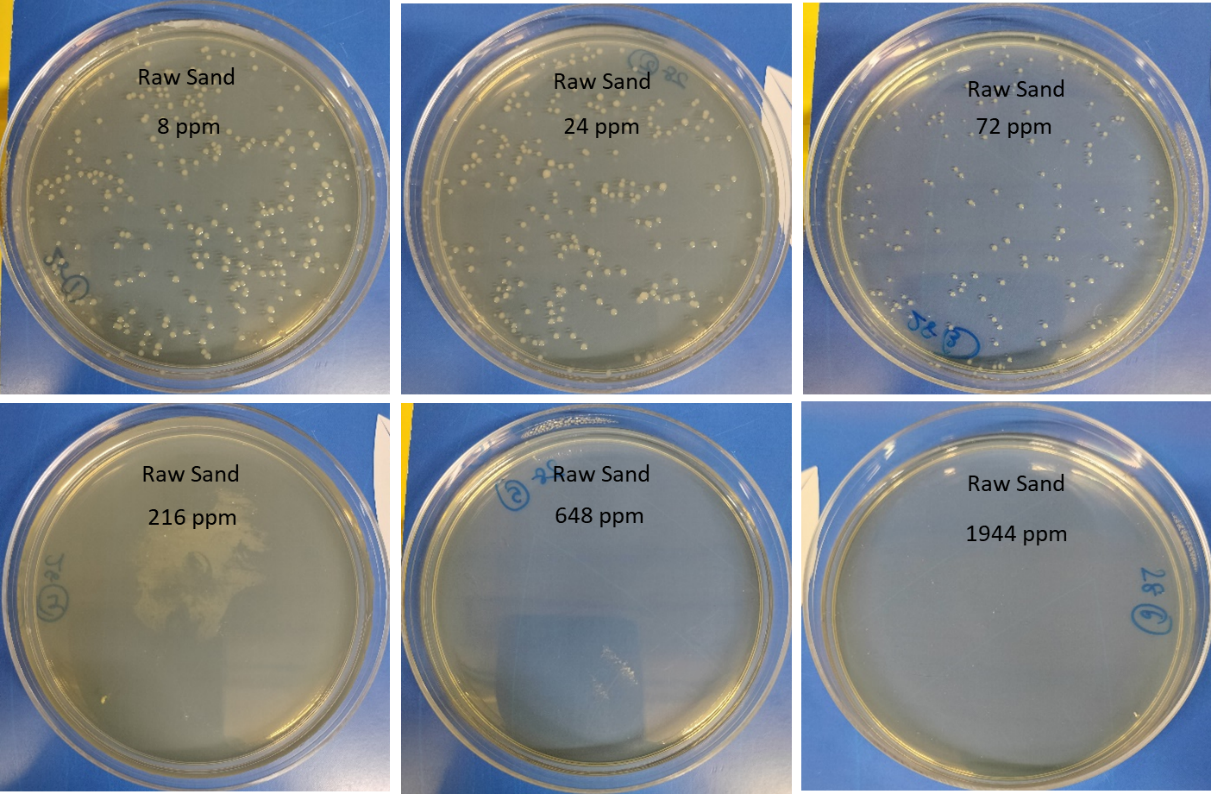
*

Fig. S6. *E. coli* with 8 ppm, 24 ppm, 72 ppm, 216 ppm, 648 ppm, and 1944 ppm of raw sand (0%Ag).

*
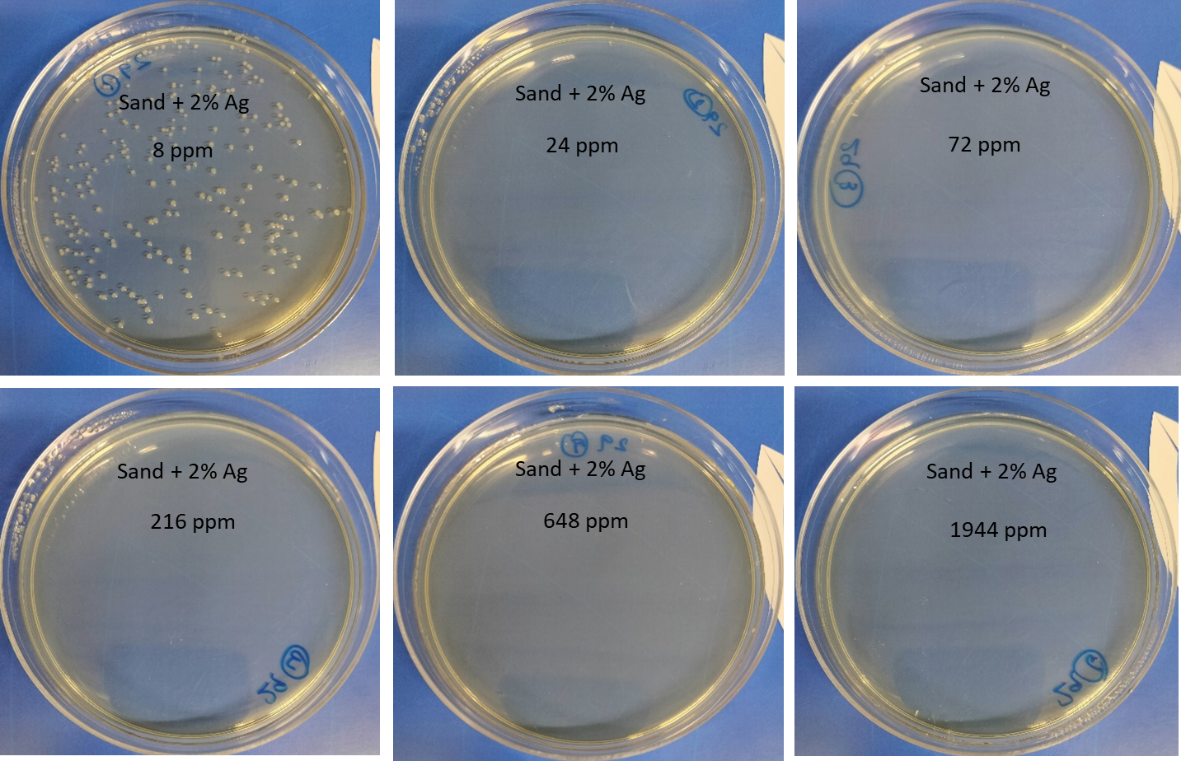
*

Fig. S7. *E. coli* with 8 ppm, 24 ppm, 72 ppm, 216 ppm, 648 ppm, and 1944 ppm of sand-coated 2% Ag nanoparticles.

*
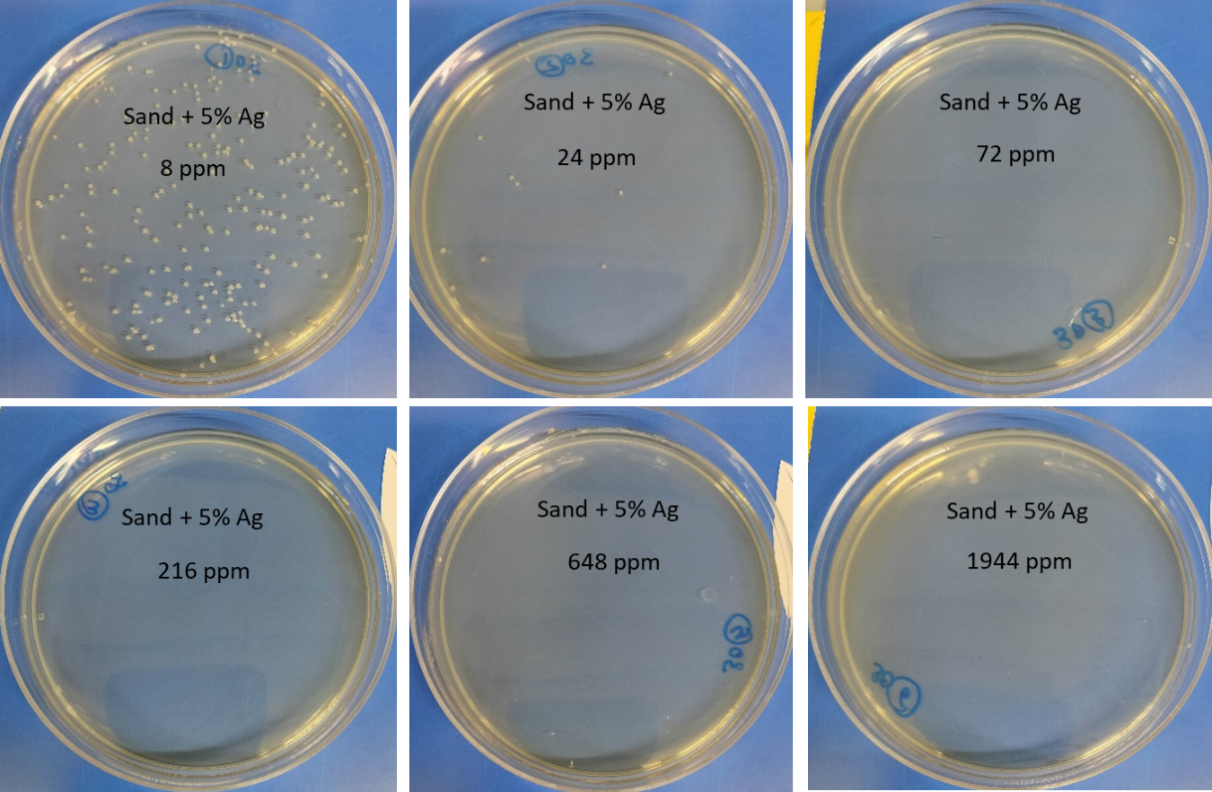
*

*Fig. S8. E. coli* with 8 ppm, 24 ppm, 72 ppm, 216 ppm, 648 ppm and 1944 ppm of sand-coated 5% Ag nanoparticles.

*
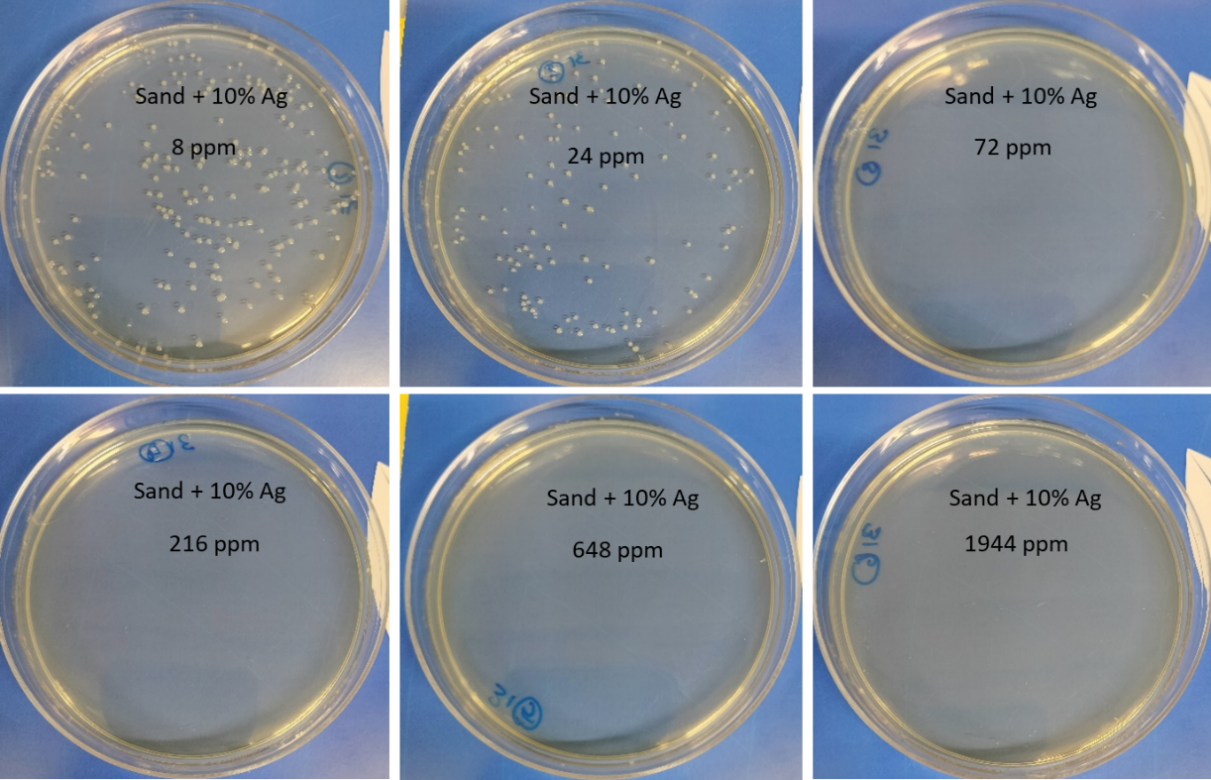
*

Fig. S9. *E. coli* with 8 ppm, 24 ppm, 72 ppm, 216 ppm, 648 ppm and 1944 ppm of sand-coated 10 % Ag nanoparticles.

| *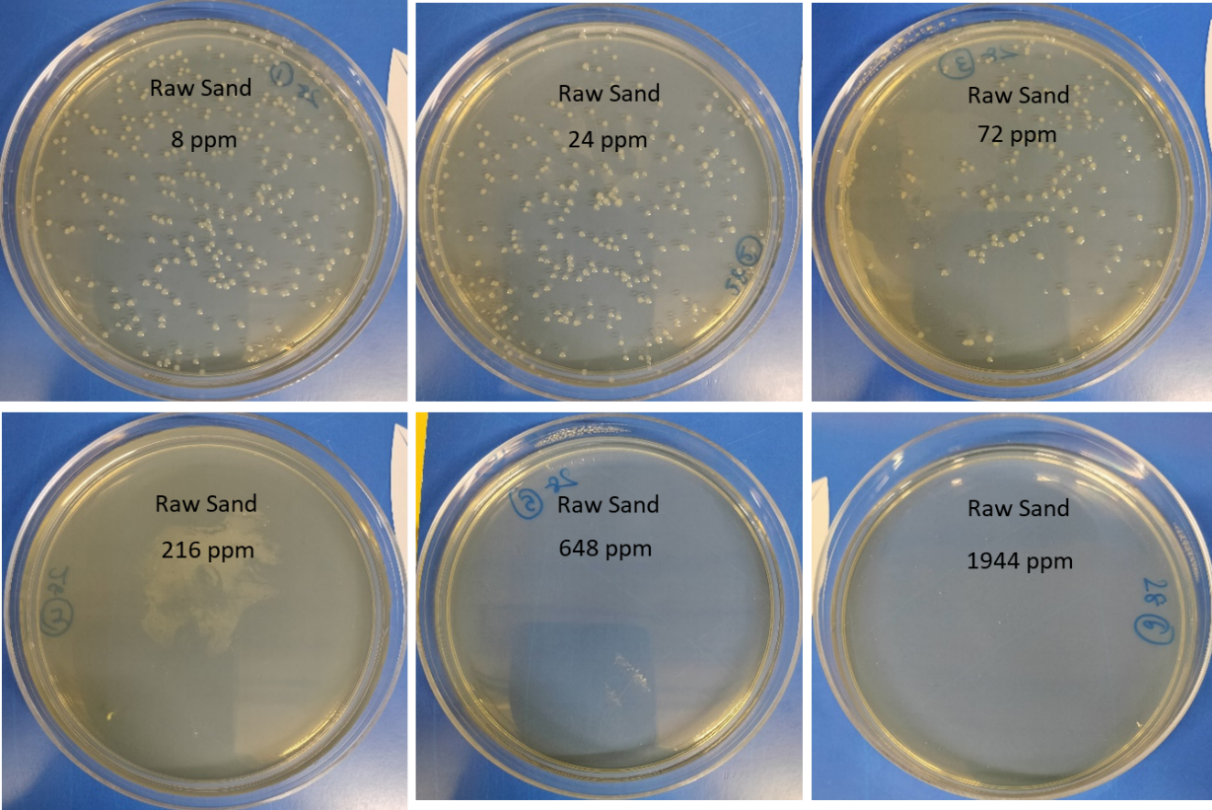* |  |  |
| --- | --- | --- |

Fig. S10. *S. aureus* with 8 ppm, 24 ppm, 72 ppm, 216 ppm, 648 ppm, and 1944 ppm of raw sand (0%Ag).

*
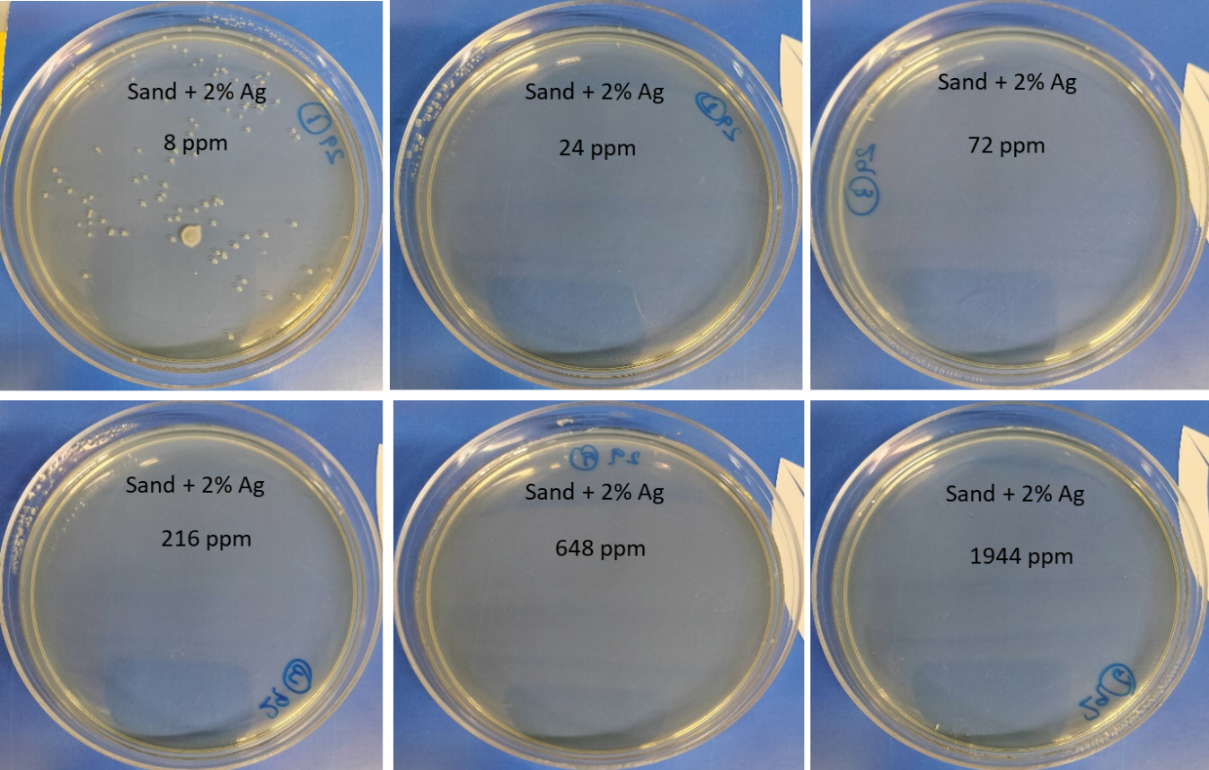
*

Fig. S11. *S. aureus* with 8 ppm, 24 ppm, 72 ppm, 216 ppm, 648 ppm, and 1944 ppm of sand-coated 2 % Ag nanoparticles.

*
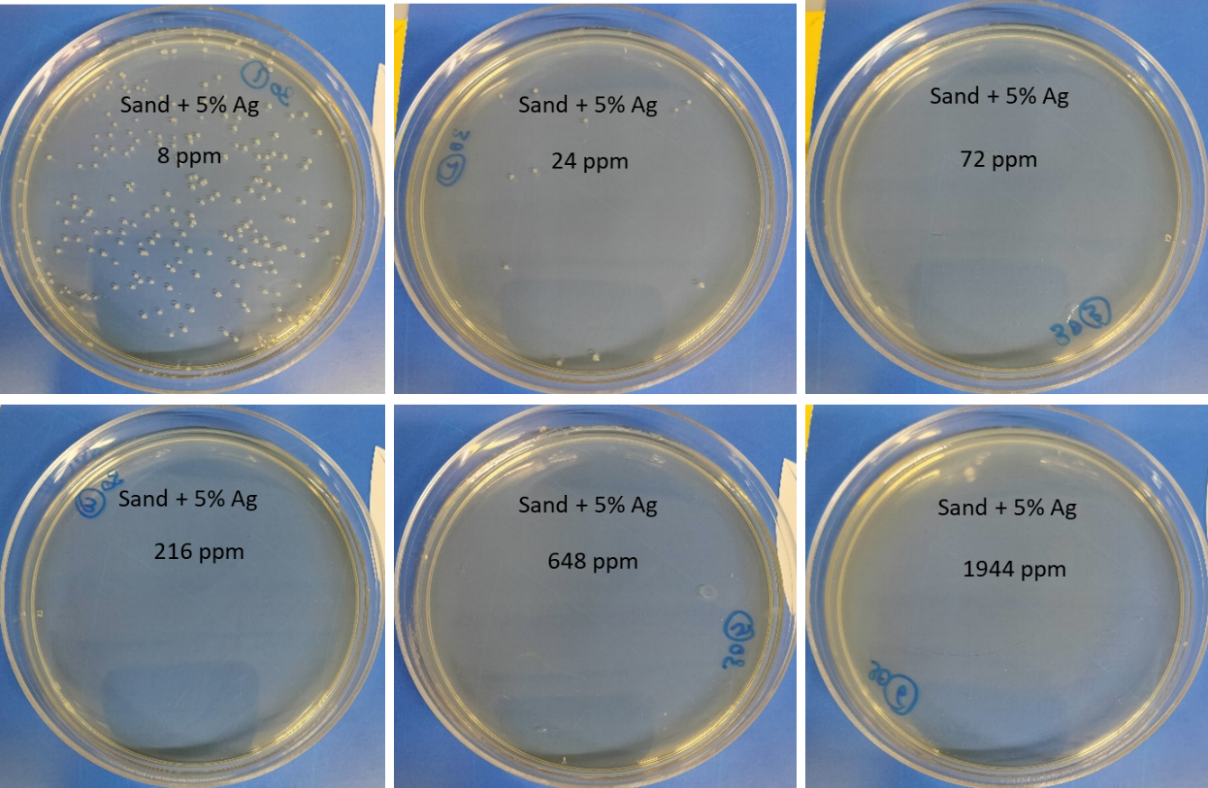
*

Fig. S12. *S. aureus* with 8 ppm, 24 ppm, 72 ppm, 216 ppm, 648 ppm, and 1944 ppm of sand-coated 5 % Ag nanoparticles.

*
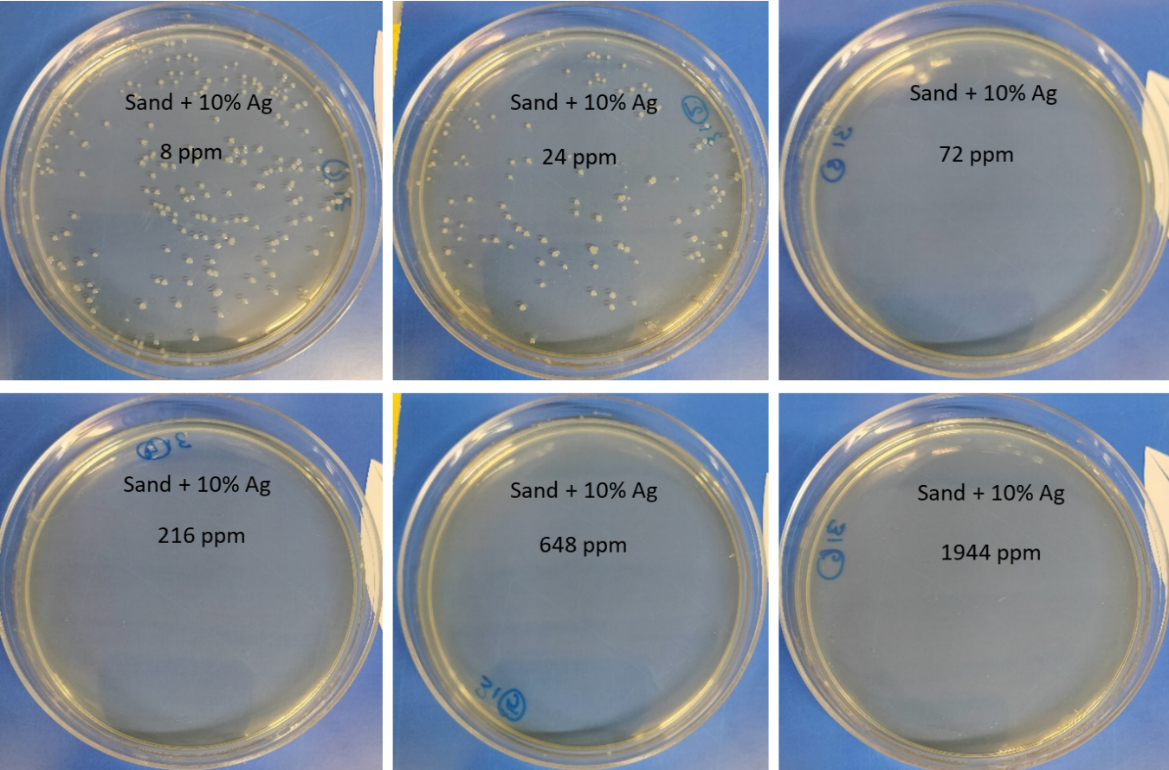
*

Fig. S13. *S. aureus* with 8 ppm, 24 ppm, 72 ppm, 216 ppm, 648 ppm, and 1944 ppm of sand-coated 10 % Ag nanoparticles.

**References**

1 Gungure, A. S., Jule, L. T., Nagaprasad, N. & Ramaswamy, K. Studying the properties of green synthesized silver oxide nanoparticles in the application of organic dye degradation under visible light. *Scientific Reports* **14**, 26967 (2024).

2 Socrates, G. *Infrared and Raman characteristic group frequencies: tables and charts*. (John Wiley & Sons, 2004).

3 Nakamoto, K. *Infrared and Raman spectra of inorganic and coordination compounds, part B: applications in coordination, organometallic, and bioinorganic chemistry*. (John Wiley & Sons, 2009).
